# Assessment of health facility capacity to provide newborn care in Bangladesh, Haiti, Malawi, Senegal, and Tanzania

**DOI:** 10.7189/jogh.07.020509

**Published:** 2017-12

**Authors:** Rebecca Winter, Jennifer Yourkavitch, Wenjuan Wang, Lindsay Mallick

**Affiliations:** 1 Department of Health, The District of Columbia, Washington DC, USA; 2 ICF, Rockville, Maryland, USA; 3 The DHS Program, ICF, Rockville, Maryland, USA; 4 The DHS Program, Avenir Health, Glastonbury, Connecticut, USA

## Abstract

**Background:**

Despite the importance of health facility capacity to provide comprehensive care, the most widely used indicators for global monitoring of maternal and child health remain contact measures which assess women’s use of services only and not the capacity of health facilities to provide those services; there is a gap in monitoring health facilities’ capacity to provide newborn care services in low and middle income countries.

**Methods:**

In this study we demonstrate a measurable framework for assessing health facility capacity to provide newborn care using open access, nationally–representative Service Provision Assessment (SPA) data from the Demographic Health Surveys Program. In particular, we examine whether key newborn–related services are available at the facility (ie, service availability, measured by the availability of basic emergency obstetric care (BEmOC) signal functions, newborn signal functions, and routine perinatal services), and whether the facility has the equipment, medications, training and knowledge necessary to provide those services (ie, service readiness, measured by general facility requirements, equipment, medicines and commodities, and guidelines and staffing) in five countries with high levels of neonatal mortality and recent SPA data: Bangladesh, Haiti, Malawi, Senegal, and Tanzania.

**Findings:**

In each country, we find that key services and commodities needed for comprehensive delivery and newborn care are missing from a large percentage of facilities with delivery services. Of three domains of service availability examined, scores for routine care availability are highest, while scores for newborn signal function availability are lowest. Of four domains of service readiness examined, scores for general requirements and equipment are highest, while scores for guidelines and staffing are lowest.

**Conclusions:**

Both service availability and readiness tend to be highest in hospitals and facilities in urban areas, pointing to substantial equity gaps in the availability of essential newborn care services for rural areas and for people accessing lower–level facilities. Together, the low levels of both service availability and readiness across the five countries reinforce the vital importance of monitoring health facility capacity to provide care. In order to save newborn lives and improve equity in child survival, not only does women’s use of services need to increase, but facility capacity to provide those services must also be enhanced.

Sustainable Development Target 3.2 aims to end the preventable death of newborns and children under age 5, with specific goals to reduce newborn deaths to less than 12 deaths per 1000 live births, and under–five deaths to less than 25 deaths per 1000 live births in all countries by 2030 [1]. Recent gains in child survival have been concentrated in the post–neonatal period, with slower gains made in survival during the first month of life [2]. As a result, the percentage of under–five deaths occurring in the first month of life has increased from 38 percent in 2000 to 45 percent in 2015 [3,4]. To continue making gains in child survival, it is essential to ensure that all newborns receive the care they need to survive.

Mothers are advised to give birth in health facilities in order to protect both their own and their infants’ health [5,6]. Interventions during labor and birth, including those addressing obstetric complications, are known to have the greatest impact on neonatal survival, followed by appropriate care for small or ill newborns [7]. Specific interventions that have an impact on neonatal mortality include umbilical cord antiseptics, neonatal resuscitation, hypothermia for hypoxic ischaemic encephalopathy, topical emollient therapy, hypothermia prevention for preterm infants, Kangaroo Mother Care in preterm infants, oral and injectable antibiotics for pneumonia, and antibiotics for sepsis [7]. While evidence from a systematic review and meta–analysis suggests that delivering in a facility reduces the overall risk of neonatal mortality in low– and middle–income countries [6], not all studies have found facility delivery to be protective for newborn survival [8–10]. In fact, several recent studies using household survey data have found no evidence that the scale–up of facility deliveries or skilled birth attendance has been associated with reductions in neonatal mortality [10–12]. The provision of newborn care in the immediate and early postnatal period is particularly dependent on health facility infrastructure, capacity, and resources [13], and delivering in a facility that is ill–equipped to provide newborn care may not protect the infant. It is critical to ensure an optimal standard of care for mothers and newborns in health facilities, yet there is a gap in monitoring the quality of newborn care [14]. This study focuses on one specific aspect of quality of care: health facility capacity to provide newborn care, which is measured with service availability and service readiness to provide newborn care services. Service availability refers to the physical presence of essential newborn care services. Service readiness refers to the presence of essential infrastructure, functioning equipment, supplies, medicines that are in–stock and non–expired, trained staff, and current guidelines to provide the services. Both are prerequisite to providing good–quality services [15].

Despite agreement on the key packages and health interventions needed to protect and save newborn lives, there is little consensus on which are the key indicators needed to assess health facilities’ capacity to provide newborn care [16]. The basic and comprehensive emergency obstetric care (EmOC) signal functions – shortlists of life–saving services first introduced in 1997 by the United Nations – are widely used to assess the functionality of health facility delivery care. But these functions focus primarily on provisions to treat the main causes of maternal mortality. With the exception of one recently added signal function on newborn resuscitation (introduced in 2009), the EmOC signal functions do not gauge facility readiness to provide essential newborn care [17]. Work has been under way to develop metrics for measuring facility provision of newborn care [15,16,18]. In 2008, Save the Children’s Saving Newborn Lives program (SNL) convened a Newborn Indicators Technical Working Group (TWG) composed of evaluation and measurement experts, researchers, UN agencies, non–governmental organizations and donors. This group collaborated to construct a list of survey–based indicators to assess whether a facility is able to address the three leading causes of newborn death: intrapartum causes (eg, birth asphyxia), preterm birth, and infection. The evidence–based list of newborn care service indicators that they developed includes measures of service availability, equipment and supplies, documentation, staff training, supervision, and additional optional indicators [17]. Gabrysch and colleagues (2012) also proposed a set of obstetric and newborn signal functions that includes four areas: general health facility requirements, routine care for all mothers and babies, basic emergency care for mothers and babies with complications, and comprehensive emergency care functions [15]. Finally, the WHO Service Availability and Readiness Assessment (SARA) includes numerous indicators on newborn care [18]. In this study we combined indicators from these three sources to generate metrics to assess the availability and readiness of labor and delivery and immediate postnatal care provided at health facilities, in light of their impact on newborn morbidity and mortality.

The USAID–funded Service Provision Assessment (SPA) survey, implemented by the Demographic Health Surveys (DHS) Program, collects nationally–representative information about health facilities’ service delivery, providing a key resource for assessing the extent to which facilities can provide comprehensive newborn care. In this study, we examined facility capacity to provide newborn care among facilities that offer delivery services in Bangladesh, Haiti, Malawi, Senegal, and Tanzania, five countries with high levels of neonatal mortality and recent SPA data. As of 2015, the neonatal mortality rates in the five countries ranged from 19 deaths per 1000 live births in Tanzania to 25 deaths per 1000 live births in Haiti, according to the UN Inter–agency Group for Child Mortality. For Senegal, Malawi, and Bangladesh, the rates were 21, 22, and 23 deaths per 1000 live births, respectively [19]. This study is the first comparative presentation of facility capacity to provide newborn care in multiple countries, using a measurable framework that could inform future studies. The manuscript originated from an earlier analysis carried out by the same authors [20] with a narrowed scope on key findings regarding newborn care service availability and readiness.

## METHODS

### Data

Study countries were selected according to two criteria. We focused the initial selection on the 25 USAID maternal and child health (MCH) priority countries (for a listing of the countries, see [21]). These countries account for more than 66% of global maternal and child deaths and are the focus of USAID programmatic efforts to scale up high–impact interventions and strengthen health systems [21]. We then restricted the analysis to countries with a SPA survey conducted within the last five years (ie, since 2011) with data available as of May 2016. Three of the five surveys included in the study are nationally representative sample surveys, while two (Haiti 2013 and Malawi 2013–14) are a census of all health facilities in the country (**Table 1**). The study was restricted to facilities that offer delivery services. Sample weights were applied throughout the study so that indicator estimates are representative of each country’s actual mix of facilities, rather than the sample’s mix of facilities. All five surveys produced indicators that are representative at the national level by facility type, managing authority, and geographic region.

**Table 1:** Description of SPA surveys included in the study

Country/y	Number of facilities*	Unweighted number of facilities with delivery services	Weighted number of facilities with delivery services	Sample or census
Bangladesh 2014	1548	586	280	sample
Haiti 2013	905	389	389	census
Malawi 2013–14	977	528	528	census
Senegal 2014†	363	282	279	sample
Tanzania 2014–15	1188	951	905	sample

*For all SPA surveys, the facility weights are normalized to have an equal unweighted and weighted total number of facilities.

†The Senegal 2014 SPA is part of the Senegal Continuous Survey project, which is designed to have five annual rounds of both DHS and SPA data collection, with the last round in 2017. This study uses the most recent available year of data, 2014. This survey included a subsample of health huts (case de santé). However, the methodology used to select health huts was different and their probability of selection was dependent on that of the health posts with which they were affiliated. Health huts are excluded from the current study.

SPA surveys provide information on the availability and readiness of health services. Specifically, the SPA surveys collect data on facility infrastructure (running water, electricity, privacy, etc.), the availability of resources (equipment, supplies, and medicines) and supportive processes and systems (client records, supervision, staff training, etc.) related to antenatal care, delivery care, and newborn care services (For more information on SPA surveys, see [22]).

SPA surveys include four standardized data collection instruments—the Facility Inventory Questionnaire, the Provider Interview Questionnaire, Observation Protocol, and Client Exit Interview—which provide general and service–specific information on the availability and quality of health services. This study relied primarily on the Facility Inventory Questionnaire, which collects information on health facilities’ infrastructure, supplies, medicines, staffing, training, and procedures, as well as on the availability of specific delivery and newborn services, through interviews with the person most knowledgeable about delivery services in the facility. The study also drew upon the Provider Interview Questionnaire, which collects information on the experience, qualifications, and perceptions of the service delivery environment among health care workers who provide selected services.

### Measurement of readiness

Our analysis focused on 38 tracer indicators to assess facilities’ capacity to provide newborn care. In order to have this capacity, a facility must (1) offer key newborn–related services, and (2) have on–site the technology, equipment, medicine, training, and knowledge required to provide those services. Thus, we assessed two dimensions of facilities’ capacity to provide newborn care: service availability and service readiness. Service availability captures the reported availability of essential newborn care services at the facility, while service readiness captures the facility’s observed capacity to provide those services [23]. We assessed three domains of service availability: the availability of basic emergency obstetric care (BEmOC) signal functions, newborn signal functions, and routine perinatal practices; and four domains of service readiness: general facility requirements, equipment, medicines and commodities, and guidelines and staffing. Table S1 in **Online Supplementary Document(Online Supplementary Document)** presents the seven domains, lists and defines the indicators, and notes their relevance to newborn health.

The 38 newborn care indicators were drawn primarily from a list of indicators suggested by the SNL TWG, and supplemented with additional WHO SARA indicators of “basic obstetric and newborn care” [18], and with Gabrysch and colleagues’ [15] proposed obstetric and newborn signal function indicators. The study did not include prevention of mother to child transmission of HIV indicators since the burden of HIV varies substantially across the study countries and HIV is not a common cause of newborn death; it becomes more relevant for the post–neonatal period [24]. Several other suggested indicators (eg, referral services for lower–level facilities) are not available in the SPA surveys, along with information on these items: resuscitation table, towel for drying the baby, or up–to–date delivery register.

In accordance with the WHO SARA approach, we computed composite indicators to assess overall newborn care service availability and readiness in the facilities. We weighted the indicators within each domain of service availability and service readiness equally to produce a domain score, and weighted each domain equally to produce a summary score for service availability and for service readiness. This simple additive scale is easily replicable.

We examined newborn care service availability and readiness nationally, as well by type of facility (hospital, health center, dispensary/clinic), managing authority (public vs private/other), urban–rural location, and region (see Section B in **Online Supplementary Document(Online Supplementary Document)**). For managing authority, the private/other category included NGOs, Mission or religious–run health facilities, and parastatal facilities. For region, the 14 regions presented in Senegal’s 2014 SPA final report were aggregated into six geographic zones to have sufficient sample size in each geographic area [25].For additional detail, refer to Tables S2a–S6g in the **Online Supplementary Document(Online Supplementary Document)** that show the individual components that comprise the seven dimensions of newborn care service availability and readiness disaggregated by facility characteristics, separately for each country.

For the three countries with sampled health facilities, we presented confidence intervals around coverage point estimates (Stata v. 14), accounting for the SPA complex sample design. For the two countries that used censuses of all formal health facilities, confidence intervals are not needed, since the point estimates describe the full population of formal health facilities.

## RESULTS

### Profile of facilities with delivery services

**Table 2** shows the percent distribution of facilities offering delivery services by facility characteristics and country. In all five countries, the majority of facilities with delivery services were in rural areas—ranging from 61% in Haiti to 85% in Tanzania and Malawi. In Bangladesh and Haiti there was a fairly even distribution of hospitals, health centers, and dispensaries or clinics. In both countries, hospitals constituted roughly one–quarter of facilities with delivery services. In Senegal and Tanzania the vast majority of facilities with delivery services were either dispensaries or clinics (89% and 83%, respectively). Malawi stands out as the only country where the majority of facilities with delivery services were health centers (78%). Between 50% and 90% of the facilities were public. The managing authorities included within “private or other” varied by country, and included private, parastatal, NGO, for profit, and religious–affiliated facilities. Haiti had the largest share of private or other facilities with delivery services (50%). In Haiti these were a mix of NGO/private not for profit, private for profit, and Mission or faith–based facilities.

**Table 2:** Percent distribution of facilities with delivery services by facility characteristics, Bangladesh, Haiti, Malawi, Senegal, Tanzania

	Bangladesh		Haiti		Malawi		Senegal		Tanzania	
	**%**	**N**	**%**	**N**	**%**	**N**	**%**	**N**	**%**	**N**
**Facility type:**										
Hospital	26.1	73	24.1	94	18.0	95	4.0	11	4.8	44
Health Center	35.2	99	42.9	167	78.5	414	7.3	20	12.1	110
Dispensary/Clinic	38.7	109	33.0	128	3.5	19	88.7	248	83.1	751
**Urban–rural:**										
Urban	29.3	82	38.8	151	14.8	78	25.9	72	14.6	132
Rural	70.7	198	61.2	238	85.2	450	74.1	207	85.4	773
**Managing authority:**										
Public	79.8	224	50.0	195	65.7	347	89.8	251	83.6	756
Private or other	20.2	57	49.8	194	34.3	181	10.2	29	16.4	149
**Total**	**100.0**	**280**	**100.0**	**389**	**100.0**	**528**	**100.0**	**279**	**100.0**	**905**

### Overall service availability and readiness

**Figure 1:**
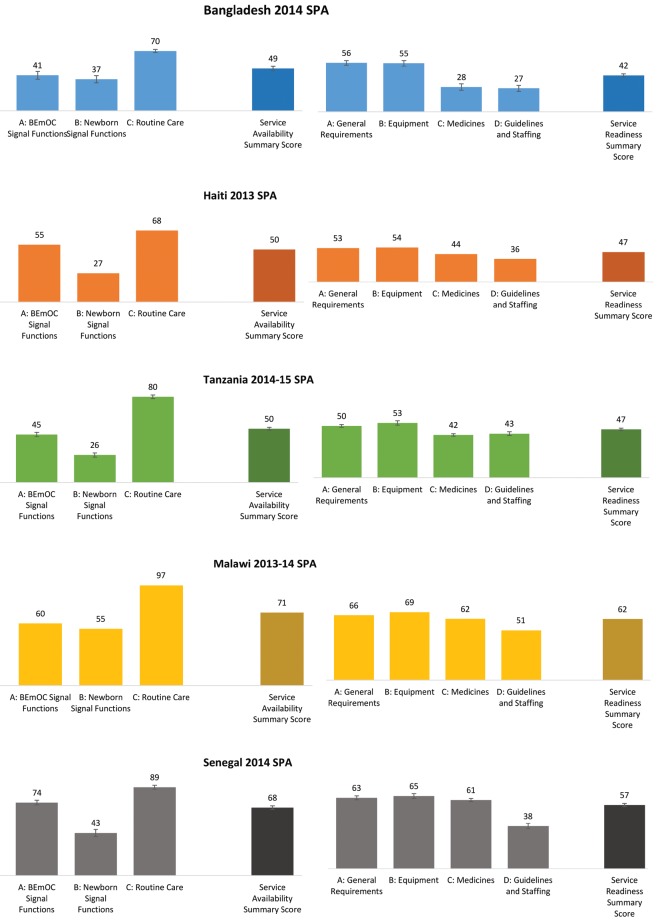
National service availability and service readiness summary scores, Bangladesh, Haiti, Tanzania, Malawi, Senegal. Confidence intervals are not shown for Haiti or Malawi, since those surveys were a census of all formal health facilities.

**Figure 1** shows national scores for each domain of availability and readiness, as well as national summary scores for service availability and service readiness. All scores range from 0 to 100 and indicate the average percentage of component tracer items that are available within the domain.

In all countries, of the three domains of service availability, scores for routine care availability were highest and scores for newborn signal function availability were lowest. Routine care scores ranged from about 70 in Bangladesh and Haiti to 97 in Malawi. This domain assessed the availability of three services: routine use of a partograph at the facility to monitor and manage labor, routine early initiation of breastfeeding, and routine thermal care, including drying and wrapping. While early initiation of breastfeeding and routine thermal care were nearly universal in each country, routine use of the partograph was less prevalent (see Tables S2a–S6g in the **Online Supplementary Document(Online Supplementary Document)**).

Scores for the newborn signal function domain ranged from 26 and 27 in Tanzania and Haiti, respectively, to 55 in Malawi. Coverage of each of the three services included in the domain—corticosteroids in preterm labor, KMC for premature/very small babies, and neonatal resuscitation—was low, with the availability of corticosteroids in preterm labor scoring lowest (see Tables S2a–S6g in the **Online Supplementary Document(Online Supplementary Document)**).

Scores for the third domain of service availability, the BEmOC signal functions, ranged from 41 in Haiti to 74 in Senegal. Of the six BEmOC functions, parenteral administration of anticonvulsants was least available in facilities, while parenteral administration of uterotonic drugs was most available (see Tables S2a–S6g in the **Online Supplementary Document(Online Supplementary Document)**). Overall, the summary scores for newborn care service availability ranged from 49 in Bangladesh to 71 in Malawi.

Coverage patterns for the four domains of service readiness were consistent across the countries. Of the four domains, scores for guidelines and staffing were lowest, followed by scores for medicines. Guidelines and staffing scores ranged from 27 in Bangladesh to 51 in Malawi. This domain included six indicators of newborn–care related staff training, three indicators on the presence of key guidelines, and one indicator of supervision. Nearly all indicators in the domain scored poorly, with the exception of staff supervision (see Tables S2a–S6g in the **Online Supplementary Document(Online Supplementary Document)**).

Scores in the medicines domain were also low, ranging from 28 in Bangladesh to about 60 in Malawi and Senegal. Of eight essential medicines in the domain, five were unavailable in more than half of facilities with delivery services in Bangladesh, Haiti, and Tanzania. These five medicines were chlorhexidine for cord cleaning, magnesium sulfate, hydrocortisone, injectable antibiotic, and antibiotic eye ointment for the newborn (see Tables S2a–S6g in the **Online Supplementary Document(Online Supplementary Document)**).

Within each country, scores for the general requirements and equipment domains were higher, and were similar to each other. General requirements scores ranged from 50 in Tanzania to 66 in Malawi. This domain included five indicators: the availability of emergency transport, 24/7 skilled birth attendance, improved sanitation, an improved water source, and electricity. Of these, 24/7 skilled birth attendance was least prevalent, followed by emergency transport and improved sanitation (see Tables S2a–S6g in the **Online Supplementary Document(Online Supplementary Document)**).

Equipment scores ranged from 53 in Tanzania to 69 in Malawi. The domain included 13 indicators, including sterilization equipment, delivery bed, examination light, delivery pack, suction apparatus, manual vacuum extractor, vacuum aspirator or D&C kit, partograph, disposable latex gloves, newborn back and mask, infant scale, blood pressure apparatus, and handwashing soap and running water or hand disinfectant. Of these, facility coverage of manual vacuum extractors and vacuum aspirator or D&C kits tended to be lowest, followed by newborn bag and masks and sterilization equipment (see Tables S2a–S6g in the **Online Supplementary Document(Online Supplementary Document)**). Overall, the summary scores for newborn care service readiness ranged from 42 in Bangladesh to 62 in Malawi.

**Figure 2:**
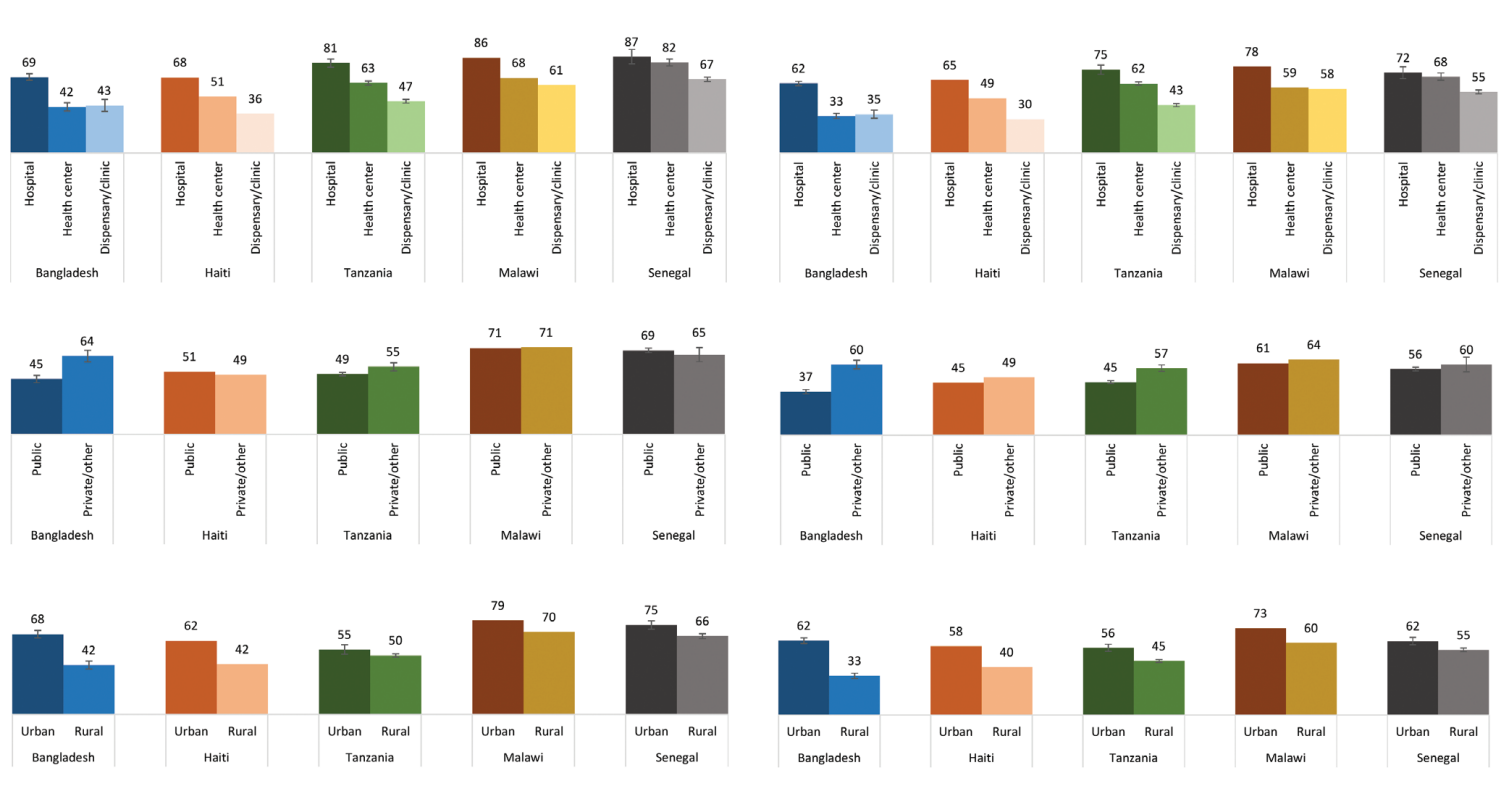
Service availability (panels on left) and service readiness (panels on right) summary scores by facility characteristics, Bangladesh, Haiti, Tanzania, Malawi, Senegal. Confidence intervals are not shown for Haiti or Malawi, since those surveys were a census of all formal health facilities.

**Figure 2** presents differentials in the composite service availability and service readiness scores side by side. The patterns in service availability and service readiness are strikingly similar across countries. Scores for both service availability and service readiness tended to be highest in hospitals and in urban areas. There was less difference in scores between public and private facilities, except for Bangladesh and Tanzania, where private facilities scored notably higher for both service availability and service readiness. To see the individual components that comprise the seven dimensions of newborn care service availability and readiness disaggregated by facility characteristics, see Tables S2a–S6g in the **Online Supplementary Document(Online Supplementary Document)**.

## DISCUSSION

Where you are born strongly affects your chance of survival [26]. Previous studies have found that access to delivery care alone is not enough to reduce early neonatal mortality rates [8]. It is essential that the facility where the birth occurs be equipped to provide key life–saving services for the newborn. Our study is the first study to examine the capacity of facilities to provide newborn care in five countries with high neonatal mortality. In all five countries, key services, commodities, and medicines needed for comprehensive delivery and newborn care were missing from a large proportion of the facilities that offer delivery services. This is important not only because it indicates a likelihood of poor quality of care, or possibly no care, but also because widespread perceptions of services of poor quality can deter women from seeking any care at a facility [27].

Of the five countries assessed, Malawi had the highest scores for both service availability and service readiness. Service availability scores in Malawi varied by domain, with the lowest scores being for BEmOC signal functions and newborn signal functions. Service readiness domain scores were generally consistent, at around 60. However, a need for improvement remains for all domains except routine care. Hospitals scored the highest for service availability and service readiness, while health centers scored much lower. Because health centers constitute nearly 80% of the facilities that provide delivery services, and they have the potential to serve more people than hospitals, further investment in the availability and readiness of their newborn care services is greatly needed. Urban facilities generally scored higher than rural facilities, and there was only a slight difference between public and private facility scores. Most facilities are located in rural areas (85%), and investments to increase the scores of those facilities in Malawi could have a significant impact on the population. Our findings are consistent with a study by Zimba and colleagues (2012) that found most facilities had staffing and supply shortages and lacked three or more signal functions [28].

Senegal’s newborn care quality scores were nearly as high as those in Malawi, at 68 points for service availability and 57 points for service readiness. Senegal’s highest score was for routine care (89); its lowest scores were for guidelines and staffing (38) and the availability of newborn signal functions (43). Compared with the other countries, disparities are markedly less drastic in Senegal. There was little difference between public and private facilities in service availability and readiness, and on average the differences between urban and rural facilities are smaller than for other countries except Malawi. Still, health posts—which constitute nearly 90% of all facilities that offer delivery services and 100% of the “dispensaries and clinics” category—scored substantially lower than hospitals. Investments to increase the scores of health posts could have a significant impact on population health.

Bangladesh showed impressive reductions in neonatal mortality between 1990 and 2015, with the NMR declining steadily and incrementally from 63 deaths per 1000 live births in 1990 to 23 deaths per 1000 live births in 2015 [19]. Despite this improvement, we found that the country had relatively low scores for newborn care service capacity, with scores below 50 for both service availability and service readiness. Its highest score was for routine care (70) and its lowest was for the availability of medicines (28). Of the five countries, Bangladesh also showed the widest gaps in coverage across subgroups: hospitals generally had much higher scores than health centers or dispensaries/clinics; private facilities had higher scores than public facilities, and urban facilities had higher scores than rural facilities, suggesting geographic and economic inequities in access to high–quality newborn care. These findings are consistent with other studies that reported inadequate quality of obstetric care in the country and marked urban–rural gaps in quality [29]. Bangladesh has been a global leader in prioritizing newborn survival and care [30], and its policy efforts have been highly successful, as evidenced by the reductions in NMR. The relatively low quality scores could be explained by the country’s newborn care policy emphasis on community health workers and home and community–based interventions [30]. That emphasis makes sense given that 62% of women in Bangladesh deliver at home, according to the 2014 Bangladesh DHS [31]. However, given that the remaining 38% of women deliver in health facilities, concentrated efforts to improve the quality of newborn care in health facilities are urgently needed, and could lead to further reductions in neonatal mortality.

Tanzania also scored around 50 for overall service availability and service readiness, with its highest score attained for routine care (80) and its lowest score for newborn signal functions (26). Hospitals, which constitute just 5% of the country’s health facilities with delivery services, had higher scores than health centers or dispensaries/clinics; private facilities had higher scores than public facilities; and urban facilities had higher scores than rural facilities, suggesting both geographic and economic inequities. While Tanzania made impressive gains in child survival between 2000 and 2015, improvements in neonatal survival were far slower [32]. Afnan–Holme and colleagues (2015) reported important differences in funding and implementation strategies among child, maternal, and newborn health policies in Tanzania that could have contributed to these different trajectories [32]. Child survival began receiving consistent policy attention in the mid–1980s, while attention to maternal health came later, in the mid–1990s, and attention to newborn care even later, in 2005. While Tanzania’s child survival policy strategy has focused primarily on implementing high–impact interventions at the first level of the health system, maternal health interventions have often been targeted at higher levels of the health system [32]. Newborn care policies are just now rapidly scaling up in Tanzania [32]. These policies should target newborn care readiness at all facility levels, with an emphasis on first level facilities where readiness is currently lowest, and where more than 30% of women deliver [33].

Haiti scored around 50 for both service availability and service readiness, with a highest score of 68 for routine care and a lowest score of 27 for newborn signal functions. Overall in Haiti, public and private facilities scored similarly, but hospitals scored higher than health centers and dispensaries/clinics, and urban facilities scored higher than rural facilities, signaling geographic inequities and probable barriers to access. These findings are consistent with those of Wang and colleagues (2014), who also found that lower–level facilities in Haiti—and specifically health centers without beds and dispensaries—are poorly prepared to provide delivery services. In Haiti, health centers without beds and dispensaries lack a government mandate to provide delivery services [34], yet these facilities constitute half of all facilities that report offering delivery care [35]. Facilities that lack an official mandate may not receive necessary support from the government. Since lower–level facilities are often the only option in rural areas, the government should formally include them as providers of delivery care and equip them with the medicines, commodities, personnel, and training necessary to provide high–quality delivery and newborn care [35].

The study has several limitations. Our choice of countries was limited by the availability of SPA surveys. With data from SPA surveys, we cannot assess all aspects of the quality of newborn care. In this study we focused on two measurable dimensions of quality: service availability and service readiness. These two dimensions are necessary—but not sufficient—components of providing high–quality newborn care. While the indicators used to measure service availability and readiness were suggested by global experts in the field, there is a lack of evidence on a few indicators (eg, recent staff training in neonatal resuscitation, use of corticosteroids in preterm labor) about their association with newborn health outcomes. Furthermore, the service availability and readiness scores we created include 38 tracer indicators and condense a great deal of information that needs to be “unpacked” for clear interpretation and program purposes. However, we believe the scores provided a valid way to summarize a large amount of related information. The strength of our study is our multifaceted analysis, through which we sought to expose the current status of two components of newborn care quality from different angles.

We conclude that facility capacity to provide newborn care is lacking in five countries with a high burden of neonatal mortality. Of the seven domains of service availability and service readiness studied, routine care consistently scored highest, while newborn signal functions and guidelines and staffing tended to score lowest. The results point to persistent inequality in access to high–quality newborn care between urban and rural areas and between hospitals and the more commonly used health centers and dispensaries/clinics. Health system initiatives to improve facility capacity are needed in each of the five countries. All facilities that offer delivery services must have trained staff available around–the–clock and be equipped with the essential supplies, medicines, and commodities needed to care for the mother–newborn dyad during labor, delivery, and the immediate postnatal period.
