# Supplementary material for: Assessment of health facility capacity to provide newborn care in Bangladesh, Haiti, Malawi, Senegal, and Tanzania
Source: J Glob Health. 2017 Dec 20;7(2):020509. doi: 10.7189/jogh.07.020509 (PMC5804038; doi:10.7189/jogh.07.020509)
Supplement: Online Supplementary Document [file jogh-07-020509-s001.pdf]

Online Supplementary Document

Winter et al. Assessment of health facility capacity to provide newborn care in Bangladesh, Haiti, Malawi, Senegal, and Tanzania

J Glob Health 2017;7:020510

A. Additional details for the method section

Table S1. Summary of metrics used to assess newborn care service availability and service readiness at health facilities

		Indicator name	Definition	Recommended as indicator by:	Importance of this component of care
Service Availability	Domain A: Basic emergency obstetric care (BEmOC) signal functions	Parenteral administration of antibiotics	Among facilities offering delivery services, percentage reporting that they performed this signal function for emergency obstetric care at least once during the three months before the assessment	NITWG; Gabrysch et al 2012; WHO SARA	Part of EmoNC, which reduces risk of intraprum-related neonatal deaths [1].
		Parenteral administration of uterotonic drugs	see above	see above	see above
		Parenteral administration of anticonvulsants for hypertensive disorders of pregnancy	see above	see above	see above
		Manual removal of placenta	see above	see above	see above
		Assisted vaginal delivery	see above	see above	see above
		Removal of retained products	see above	see above	see above
	Domain B: Newborn signal functions	Neonatal resuscitation	Among facilities offering delivery services, percentage reporting that they performed neonatal resuscitation at least once during the three months before the assessment	NITWG; Gabrysch et al 2012; WHO SARA	Reduces risk of intrapartum-related death [1].
		Corticosteroids in preterm labor	Among facilities offering delivery services, percentage reporting that they performed this intervention at least once during the three months before the assessment	NITWG; Gabrysch et al 2012; WHO SARA	Reduces risk of neonatal death, NICU admission, and other unfavorable outcomes [1]; other evidence suggests this practice could increase neonatal mortality [2].
		KMC for premature/very small babies[1]	Among facilities offering delivery services, percentage reporting that they provide KMC for low birth weight babies	NITWG; Gabrysch et al 2012; WHO SARA	Reduces risk of neonatal mortality, hypothermia, and other unfavorable outcomes; associated with increase weight gain [1].
	Domain C: Routine perinatal practices	Partograph routinely used to monitor and manage labor[2]	Providers at the facility routinely use partograph to monitor and manage labor	Gabrysch et al 2012; WHO SARA	No evidence of effect [1]. 2014).
		Routine early initiation of breastfeeding[2]	Providers at the facility routinely initiate breastfeeding within the first hour	Gabrysch et al 2012; WHO SARA	Reduces neonatal mortality [3]; prevents diarrhea [1].
		Routine thermal care (drying and wrapping)[2]	Providers at the facility routinely dry and wrap newborns to keep them warm	Gabrysch et al 2012; WHO SARA	Wrapping newborn reduces risk of hypothermia [1].

(Continues…)

Table S1–*Continued*

		Indicator name	Definition	Recommended as indicator by:	Importance of this component of care
**Service Readiness**	**Domain A: General requirements**	Electricity	Facility is connected to a central power grid and there has not been an interruption in power supply lasting for more than two hours at a time during normal working hours in the seven days before the assessment, *or* the facility had a functioning generator with fuel available on the day of the assessment, or else facility has a back-up solar power	Gabrysch et al 2012	Light and temperature control are required for optimal care. Some pieces of equipment (e.g., ultrasound machines and incubators) require electricity to function. SARA manual: electricity for lights and communication (at a minimum) during normal working hours.
		Improved water source	Facility has an improved water source available. For most countries, this means that water is piped into the facility or onto facility grounds, or else water comes from a public tap or standpipe, a tube well or borehole, a protected dug well, protected spring, rain water, or bottled water and the outlet from this source is within 500 meters of the facility	Gabrysch et al 2012 (i.e. reliable water source)	For hygiene, (e.g., handwashing [4]), surgery [5], infection control, and drinking. Handwashing prevents neonatal tetanus and sepsis, among other infections [1].
		Improved sanitation	Facility has a functioning flush or pour-flush toilet, a ventilated improved pit latrine, or composting toilet	Gabrysch et al 2012 (i.e. clean toilets)	For human waste disposal. Poor sanitation causes unnecessary sickness and death from polluted water, food, and soil and can lead to diarrhea and other problems [6]. Diarrhea is a leading cause of death for children under age 5 [7].
		Skilled birth attendance available 24/7	Provider of delivery care available on-site or on-call 24 hours/day, with observed duty schedule	NITWG; Gabrysch et al 2012	Skilled birth attendance is fundamental to reducing maternal and neonatal mortality [8], and babies can be born at any time, any day.
		Emergency transport	Facility had a functioning ambulance or other vehicle for emergency transport that was stationed at the facility and had fuel available on the day of the assessment, or the facility has access to an ambulance or other vehicle for emergency transport that is stationed at another facility or that operates from another facility	WHO SARA	Delay in accessing care is a major risk factor for maternal and neonatal mortality. Distance to a facility and lack of transportation limit access to EmOC [9,[10,[11].

(Continues…)

Table S1–*Continued*

		Indicator name	Definition	Recommended as indicator by:	Importance of this component of care
Service Readiness	Domain B: Equipment	Sterilization equipment	Facility reports that some instruments are processed in the facility and the facility has a functioning electric dry heat sterilizer, a functioning electric autoclave, or a non-electric autoclave with a functioning heat source available somewhere in the facility	WHO SARA	Sterilization can prevent infection, a leading cause of neonatal death.
		Delivery bed	At least one delivery bed available and observed the in delivery area	WHO SARA	Provides comfort to mother and a place for her to recover from delivery.
		Examination light	Examination light (flashlight okay) available, observed, and functioning in the delivery area	WHO SARA	Providers must be able to see what they are doing.
		Delivery pack	Delivery pack OR cord clamp, episiom scissors, scissors/blade to cut cord, suture material with needle, and needle holder all available in the delivery area	WHO SARA	Provision of clean birth kits and education on their use reduces risk of neonatal mortality, perinatal mortality, and infection, and promotes sterile cord cutting [1].
		Suction apparatus (mucus abstractor)	Suction apparatus (mucus abstractor) available, observed, and functioning in the delivery area	WHO SARA	Clears infant airways to promote breathing.
		Manual vacuum extractor	Manual vacuum extractor available, observed, and functioning in the delivery area	WHO SARA	Part of EmoNC, which reduces risk of intrapartum-related neonatal deaths [1].
		Vacuum aspirator or D&C kit	Vacuum aspirator or D&C kit available, observed, and functioning in the delivery area	WHO SARA	Removes uterine contents after spontaneous or induced abortion, with a low rate of infection [12].
		Partograph	Partograph available and observed	WHO SARA	No evidence of effect [1].
		Disposable latex gloves	Disposable latex gloves observed in the delivery area	WHO SARA	Reduces risk of infection [13].
		Newborn bag and mask	Newborn bag and mask (AMBU bag and mask) available, observed, and functioning in the delivery area	NITWG; WHO SARA	Used for manual ventilation; components of supportive care package for preterm infants [1].
		Infant scale	Infant scale observed and functioning in the delivery area	NITWG; WHO SARA	Used to weigh infant; low birthweight triggers interventions.
		Blood pressure apparatus (digital or manual)	Manual or digital blood pressure apparatus observed and functioning in the delivery area	WHO SARA	Used to monitor maternal blood pressure. High blood pressure indicates the need for intervention. It may also predict early postpartum pre-eclampsia [14].
		Handwashing soap and running water or hand disinfectant	Handwashing soap and running water or hand disinfectant available and observed in the delivery area	NITWG; WHO SARA	Handwashing prevents neonatal tetanus and sepsis, among other infections [1].

(Continues…)

Table S1–*Continued*

		Indicator name	Definition	Recommended as indicator by:	Importance of this component of care
Service Readiness	Domain C: Medicines and commodities	Injectable antibiotic[3]	Injectable antibiotics observed in the delivery area and at least one dose valid	NITWG; Gabrysch et al 2012; WHO SARA	Prevents and treats infection in the infant and/or mother.
		Hydrocortisone available at the facility	Hydrocortisone observed at the facility and at least one dose valid	NITWG; Gabrysch et al 2012	Used to treat hypotension in preterm infants but is not universally advocated [15].
		Injectable uterotonic	Oxytocin observed in the delivery area and at least one dose valid	NITWG; Gabrysch et al 2012; WHO SARA	Part of EmoNC, which reduces risk of intrapartum-related neonatal deaths [1].
		Skin disinfectant	Skin disinfectant available in the delivery area	WHO SARA	Neonatal chlorhexine body wipes reduce risk of skin infection in first week of life, but not neonatal sepsis or mortality [1].
		Magnesium sulfate	Magnesium sulphate available in the delivery area and at least one dose valid	NITWG; Gabrysch et al 2012; WHO SARA	Reduces risk of eclampsia [1].
		IV solution with infusion set	IV solution with infusion set available in the delivery area and at least one set valid	Gabrysch et al 2012; WHO SARA	Used for (re)hydration.
		Chlorhexidine for cord cleaning[4]	Chlorhexidine solution (4%) for umbilical cord cleaning available in delivery area and at least one dose valid	Gabrysch et al 2012 suggested including "infection prevention including hygienic cord care" as a routine practice	Associated with reduced risk of all-cause mortality when applied to cord in first 24 hours [1].
		Antibiotic eye ointment for newborn	Tetracycline eye ointment for newborn available in delivery area and at least one dose valid	WHO SARA	Prevents newborn eye infections.

(Continues…)

Table S1–*Continued*

		Indicator name	Definition	Recommended as indicator by:	Importance of this component of care
Service Readiness	Domain D: Guidelines and Staffing	Guidelines: Integrated Management of pregnancy and childbirth (IMPAC)	Guidelines available in the delivery area	NITWG	WHO guidelines that address factors related to access to skilled care before, during and after pregnancy and birth.
		Guidelines: CEmOC	Guidelines available in the delivery area	NITWG	Guides provision of comprehensive emergency obstetric care.
		Guidelines: Management of pre-term labor	Guidelines available in delivery area.	NITWG	Guides management of pre-term labor.
		Training in neonatal resuscitation	At least one provider of delivery/newborn care in the facility received training in neonatal resuscitation in the past 24 months	NITWG; WHO SARA	Neonatal resuscitation reduces risk of intrapartum-related death [1].
		Training in early and exclusive breastfeeding	At least one provider of delivery/newborn care in the facility received training in early and exclusive breastfeeding in the past 24 months	NITWG	Early and exclusive breastfeeding prevents diarrhea [1], reduces infants' risks of respiratory illness and other infections [16,[17], and may set stage for continued breastfeeding, which has immediate and long-term benefits for mothers and children.
		Training in newborn infection management (including injectable antibiotics)	At least one provider of delivery/newborn care in the facility received training in newborn infection management (including injectable antibiotics) in the past 24 months	NITWG	Infection is a major cause of neonatal mortality [1].
		Training in thermal care	At least one provider of delivery/newborn care in the facility received training in thermal care in the past 24 months	NITWG	Hypothermia is a major risk factor for neonatal mortality, especially for small infants [1].
		Training in cord care	At least one provider of delivery/newborn care in the facility received training in cord care in the past 24 months	NITWG	Proper cord care reduces the risk of infection, which is a major cause of neonatal mortality.
		Training in KMC	At least one provider of delivery/newborn care in the facility received training in KMC in the past 24 months	NITWG	KMC reduces risk of neonatal mortality, hypothermia, and other unfavorable outcomes; associated with increase weight gain [1].
		Supervision	Percentage of facilities with routine personal supervision (at least half of interviewed providers reported being personally supervised at least once during the six months preceding the survey	NITWG	Supportive supervision can improve health worker performance of newborn care [18].

Note: Indicators in this report draw from three sources. The primary source is a list of Newborn Care Service Indicators selected by the Newborn Indicators Technical Working Group. These indicators are supplemented by additional indicators in the WHO SARA indicators of "Basic obstetric and newborn care," and by Gabrysch and colleagues' (2012) proposed obstetric and newborn signal functions. The table specifies the source of each indicator included in the analysis.

[1] Unlike the indicators for neonatal resuscitation and corticosteroids in preterm later, we do not have information on whether KMC for premature/very small babies was carried out in the last 3 months. The KMC indicator is based on the following SPA question: "Does this facility practice Kangaroo Mother Care for low birth weight babies?"
[2] We also do not have information on whether the three routine perinatal care indicators were carried out in the last 3 months. The partograph indicator is based on the following two SPA questions: "Do providers of delivery services in this facility use partograph to monitor labor and delivery" and "Are partographs used routinely (for all cases) or selectively (only for some cases) to monitor labor and delivery in this facility?". The other two indicators (drying and wrapping newborns to keep them warm, and initiation of breastfeeding within the first hour) are based on the following SPA question: "Does this facility routinely observe any of the following postpartum or newborn related practices?"
[3] The SPA surveys included in this study did not collect information on the type or brands of injectable antibiotic available in the delivery area.
[4] This indicator is assessed for all study countries, regardless of whether the country has a policy of CHX application.

References

1 Bhutta ZA, Das JK, Bahl R, Lawn JE, Salam RA, Paul VK, et al. Can available interventions end preventable deaths in mothers, newborn babies, and stillbirths, and at what cost? Lancet (London, England). 2014;384:347-70.

2 Althabe F, Belizan JM, McClure EM, Hemingway-Foday J, Berrueta M, Mazzoni A, et al. A population-based, multifaceted strategy to implement antenatal corticosteroid treatment versus standard care for the reduction of neonatal mortality due to preterm birth in low-income and middle-income countries: the ACT cluster-randomised trial. Lancet (London, England). 2015;385:629-39.

3 Debes AK, Kohli A, Walker N, Edmond K, Mullany LC. Time to initiation of breastfeeding and neonatal mortality and morbidity: a systematic review. BMC public health. 2013;13 Suppl 3:S19.

4 WHO. Service Availability and Readiness Assessment (SARA): Indicators and questionnaire. 2016. Available: http://www.who.int/healthinfo/systems/sara_indicators_questionnaire/en/. Accessed: Day Month.

5 Chawla SS, Gupta S, Onchiri FM, Habermann EB, Kushner AL, Stewart BT. Water availability at hospitals low- and middle-income countries: implications for improving access to safe surgical care.

6 Hesperian Helath Guides. Toilets for Helath (Sanitation). 2014. Available: http://en.hesperian.org/hhg/New_Where_There_Is_No_Doctor:Toilets_for_Health_(Sanitation). Accessed: Day Month.

7 WHO. Children: reducing mortality. 2016. Available: http://www.who.int/mediacentre/factsheets/fs178/en/. Accessed: Day Month.

8 Darmstadt GL, Kinney MV, Chopra M, Cousens S, Kak L, Paul VK, et al. Who has been caring for the baby? 2014;384:174-88.

9 Garenne M, Mbaye K, Bah MD, Correa P. Risk factors for maternal mortality: a case-control study in Dakar hospitals (Senegal). African journal of reproductive health. 1997;1:14-24.

10 Lema VM. Maternal and newborn health and emergency transport in Sub-Saharan Africa nd.

11 Menendez C, Romagosa C, Ismail MR, Carrilho C, Saute F, Osman N, et al. An autopsy study of maternal mortality in Mozambique: the contribution of infectious diseases. PLoS medicine. 2008;5:e44.

12 Association of Reproductive Health Professionals (ARHP). A Quick Reference Guide for Clinicians: Manual Vacuum Aspiration. 2008. Available: www.arhp.org/guide. Accessed: Day Month.

13 Ng P, Wong H, Lyon D, So K, Liu F, Lam R, et al. Combined use of alcohol hand rub and gloves reduces the incidence of late onset infection in very low birthweight infants. 2004;89:F336-F40.

14 Cohen J, Vaiman D, Sibai BM, Haddad B. Blood pressure changes during the first stage of labor and for the prediction of early postpartum preeclampsia: a prospective study. European journal of obstetrics, gynecology, and reproductive biology. 2015;184:103-7.

15 Johnson PJ. Hydrocortisone for Treatment of Hypotension in the Newborn. Neonatal network : NN. 2015;34:46-51.

16 Ladomenou F, Moschandreas J, Kafatos A, Tselentis Y, Galanakis E. Protective effect of exclusive breastfeeding against infections during infancy: a prospective study. 2010.

17 Bachrach VR, Schwarz E, Bachrach LR. Breastfeeding and the risk of hospitalization for respiratory disease in infancy: a meta-analysis. Archives of pediatrics & adolescent medicine. 2003;157:237-43.

18 Arya G. RW1-2.1 Reducing infant mortality through improved supportive supervision under IM(N)CI: Experiences from India. 2011;65:A66.

B. Health facility capacity to provide newborn care analysis

Table S2a. Service availability Domain A: BEmOC signal functions, Bangladesh 2014 SPA

	Parenteral administration of antibiotics			Parenteral administration of uterotonic drugs			Parenteral administration of anti-convulsants			Manual removal of placenta			Assisted vaginal delivery			Removal of retained products			*Service availability Domain A summary score*			
	**%**	**LB**	**UB**	**%**	**LB**	**UB**	**%**	**LB**	**UB**	**%**	**LB**	**UB**	**%**	**LB**	**UB**	**Mean**	**LB**	**UB**	**Mean**	**LB**	**UB**	**N**
**Facility type**																						
Hospital	87.9	82.3	92.0	85.8	80.1	90.1	60.7	53.5	67.5	74.2	67.5	80.0	76.1	69.5	81.7	64.6	57.4	71.2	74.9	70.8	79.0	73
Health Center	33.8	24.6	44.5	40.9	31.1	51.6	16.4	10.8	24.1	27.5	19.4	37.3	39.3	29.6	50.0	19.2	12.7	27.9	29.5	23.8	35.2	99
Dispensary or clinic	25.8	15.9	39.1	35.4	24.0	48.7	21.4	12.2	34.8	34.1	20.6	50.8	36.8	22.5	53.8	22.4	13.1	35.6	29.3	19.9	38.7	108
**Managing authority**																						
Public	36.0	29.1	43.4	43.7	36.4	51.3	24.4	18.8	31.0	36.4	28.5	45.2	44.2	35.4	53.3	26.2	20.4	33.0	35.1	30.0	40.3	224
Private or other	80.1	70.4	87.2	77.4	67.4	85.0	51.9	41.5	62.1	65.4	54.9	74.5	62.7	52.2	72.1	56.3	45.6	66.4	65.6	58.3	72.9	56
**Location**																						
Urban	84.1	77.5	89.0	81.2	74.3	86.6	55.6	48.0	62.9	73.5	66.2	79.7	71.2	63.9	77.5	63.2	55.7	70.2	71.5	66.7	76.3	82
Rural	28.6	21.2	37.3	37.7	29.7	46.5	19.3	13.3	27.1	29.3	20.7	39.6	38.3	28.7	48.8	19.4	13.3	27.5	28.8	23.0	34.6	198
**Division**																						
Barisal	47.6	35.0	60.5	46.4	33.8	59.5	19.5	11.4	31.3	45.4	32.9	58.5	41.7	29.5	55.1	29.8	20.8	40.7	38.4	30.6	46.1	15
Chittagong	53.0	40.7	64.9	51.9	39.7	63.9	27.0	18.9	36.9	46.3	34.4	58.6	47.3	34.2	60.7	32.8	22.8	44.6	43.0	33.1	53.0	58
Chaka	49.3	35.5	63.1	51.0	37.2	64.7	27.5	16.9	41.6	39.0	26.4	53.3	48.8	33.2	64.7	29.6	22.7	37.5	40.9	31.9	49.9	97
Khulna	41.5	25.6	59.5	57.6	37.2	75.8	34.4	18.8	54.2	37.5	23.3	54.3	42.7	24.7	62.9	29.0	17.5	44.0	40.4	30.2	50.7	25
Rajshahi	31.0	22.9	40.4	30.3	22.1	39.9	20.8	15.6	27.1	42.0	21.7	65.4	48.2	26.3	70.7	32.3	14.7	56.9	34.1	21.6	46.6	35
Rangpur	38.2	26.5	51.4	69.2	54.1	81.0	53.8	41.3	65.9	46.3	24.1	70.1	44.8	32.1	58.2	44.5	22.6	68.7	49.5	37.7	61.2	33
Sylhet	36.7	22.2	54.1	41.8	25.8	59.7	29.0	15.4	47.9	43.9	25.9	63.6	63.3	39.8	81.8	29.0	17.4	44.2	40.6	26.5	54.7	18
Total	44.8	38.8	51.0	50.5	44.2	56.7	29.9	24.8	35.6	42.3	35.4	49.5	47.9	40.5	55.5	32.3	27.0	38.0	41.3	36.8	45.7	280

Note: LB and UB refer to the lower and upper bounds of the 95% confidence interval.

Table S2b. Service availability Domain B: Newborn signal functions, Bangladesh 2014 SPA

	Neonatal resuscitation			Cortisosteroids for pre-term labor			Kangaroo Mother Care			*Service availability Domain B summary score*			
	%	LB	UB	%	LB	UB	%	LB	UB	Mean	LB	UB	N
**Facility type**													
Hospital	69.4	62.5	75.5	47.7	40.8	54.7	57.1	50.1	63.8	58.1	53.4	62.8	73
Health Center	31.6	22.8	42.1	10.6	6.2	17.5	34.6	25.2	45.4	25.6	20.2	31	99
Dispensary or clinic	34.9	21.1	51.9	18.8	8.9	35.4	44	27.9	61.4	32.6	23.8	41.3	108
**Managing authority**													
Public	37.9	29.7	47	18.8	13	26.4	41.5	32.4	51.2	32.7	27.9	37.5	224
Private or other	61.8	51.3	71.3	41.9	32.1	52.4	54.6	44.5	64.2	52.8	45.6	59.9	56
**Location**													
Urban	68.1	60.9	74.5	47.9	40.6	55.2	56	48.7	63.1	57.3	52.5	62.1	82
Rural	32.2	23.3	42.7	13.3	7.4	22.7	39.2	29.1	50.2	28.2	22.8	33.7	198
**Division**													
Barisal	31.7	21.4	44.2	15.4	9.4	24	35.6	24.1	49.2	27.6	20	35.1	15
Chittagong	39.2	28.5	51	15.5	9.4	24.5	41.3	29.5	54.2	32	23.7	40.2	58
Chaka	47.1	31.8	63	29.9	18.9	43.7	48.2	32.5	64.3	41.7	33.3	50.1	97
Khulna	57.7	36.8	76.1	27.6	13.3	48.6	48.4	28.7	68.7	44.6	31.1	58.1	25
Rajshahi	29.6	21.1	39.9	18.5	12.6	26.4	37.6	18.5	61.5	28.6	19.8	37.3	35
Rangpur	49.8	26.4	73.2	27.3	10.4	55	47.1	24.3	71.1	41.4	30.1	52.6	33
Sylhet	31.8	20.3	46	17.6	8.8	32.3	39.6	24.7	56.6	29.7	18	41.3	18
Total	42.8	35.7	50.1	23.4	18.2	29.7	44.1	36.4	52.1	36.8	32.6	40.9	280

Note: LB and UB refer to the lower and upper bounds of the 95% confidence interval.

Table S2c. Service availability Domain C: Routine perinatal care, Bangladesh 2014 SPA

	Partograph routinely used to monitor labor			Routine early initiation of breastfeeding (w/in first hour)			Thermal care			*Service availability Domain C summary score*			
	%	LB	UB	%	LB	UB	%	LB	UB	Mean	LB	UB	N
**Facility type**													
Hospital	24.3	18.8	30.7	97.7	94.9	98.9	96.6	93.8	98.2	72.9	70.6	75.1	73
Health Center	21.6	14.0	32.0	94.5	90.4	96.9	93.4	88.3	96.4	69.9	66.1	73.6	99
Dispensary or clinic	8.0	5.4	11.8	99.3	95.3	99.9	94.4	82.4	98.4	67.2	64.5	69.9	108
**Managing authority**													
Public	14.4	10.6	19.2	96.9	94.8	98.1	94.1	89.2	96.9	68.4	66.3	70.6	224
Private or other	27.7	20.4	36.2	98.4	95.3	99.5	96.7	91.6	98.8	74.3	71.3	77.3	56
**Location**													
Urban	28.5	22.9	34.8	98.3	96.1	99.2	96.1	92.7	98.0	74.3	72.0	76.6	82
Rural	12.3	8.3	17.9	96.7	94.4	98.1	94.0	88.3	97.0	67.7	65.3	70.0	198
**Division**													
Barisal	11.6	6.3	20.3	92.0	78.5	97.3	90.4	78.0	96.1	64.7	58.8	70.5	15
Chittagong	12.5	7.1	21.1	97.7	95.0	99.0	91.5	69.8	98.1	67.2	62.4	72.1	58
Chaka	22.5	15.4	31.8	100.0			98.7	95.6	99.6	73.8	70.9	76.6	97
Khulna	24.8	12.5	43.3	98.8	91.8	99.8	98.5	92.6	99.7	74.0	68.8	79.2	25
Rajshahi	8.9	4.4	16.9	100.0			96.5	78.5	99.5	68.5	65.4	71.5	35
Rangpur	17.7	9.4	30.8	91.0	79.3	96.4	87.7	76.2	94.1	65.5	58.7	72.3	33
Sylhet	10.7	5.8	18.9	88.0	73.3	95.1	89.6	71.3	96.8	62.8	56.7	68.8	18
Total	17.1	13.6	21.2	97.2	95.5	98.2	94.6	90.7	97.0	69.6	67.8	71.4	280

Note: LB and UB refer to the lower and upper bounds of the 95% confidence interval.

Table S2d. Service readiness Domain A: General requirements, Bangladesh 2014 SPA

	Electricity			Improved water source			Improved sanitation			24/7 skilled birth attendance			Emergency transport			*Service readiness Domain A summary score*			
	**%**	**LB**	**UB**	**%**	**LB**	**UB**	**%**	**LB**	**UB**	**%**	**LB**	**UB**	**%**	**LB**	**UB**	**Mean**	**LB**	**UB**	**N**
**Facility type**																			
Hospital	85.7	80.9	89.5	96.6	92.9	98.4	90.2	85.5	93.5	74.2	67.4	80.0	78.5	72.0	83.8	85.1	82.8	87.3	73
Health Center	38.5	29.1	48.9	93.4	87.4	96.6	73.2	62.9	81.6	12.6	8.6	18.1	13.6	8.2	21.8	46.3	42.9	49.6	99
Dispensary or clinic	30.4	22.1	40.2	89.9	69.3	97.2	65.7	46.1	81.1	17.5	10.8	27.1	19.1	10.1	33.2	44.5	37.4	51.7	108
**Managing authority**																			
Public	35.8	29.7	42.3	91.9	82.9	96.3	70.5	60.0	79.3	24.1	20.0	28.6	24.7	19.1	31.4	49.4	45.6	53.1	224
Private or other	94.9	87.9	97.9	96.8	91.0	98.9	91.5	84.7	95.4	56.4	45.9	66.4	64.1	53.8	73.3	80.7	76.9	84.6	56
**Location**																			
Urban	91.1	86.9	94.1	96.7	93.1	98.5	91.5	86.9	94.6	72.8	65.4	79.1	76.2	69.2	82.0	85.7	83.3	88.0	82
Rural	29.6	23.0	37.3	91.3	81.0	96.2	67.8	56.0	77.7	13.1	9.0	18.6	14.6	8.9	23.0	43.3	39.0	47.5	198
**Division**																			
Barisal	40.1	29.0	52.3	88.8	76.4	95.1	77.7	62.7	87.8	39.5	29.6	50.3	32.3	26.0	39.4	55.7	50.7	60.7	15
Chittagong	51.8	41.3	62.2	95.9	88.0	98.7	86.8	68.9	95.1	28.7	20.3	38.9	32.6	24.6	41.7	59.2	53.4	64.9	58
Chaka	45.4	38.2	52.7	90.4	70.4	97.4	68.2	50.3	81.9	28.3	23.4	33.7	37.2	24.9	51.4	53.9	47.3	60.4	97
Khulna	62.7	44.5	77.9	97.6	91.1	99.4	54.7	32.9	74.8	34.0	25.0	44.3	45.4	28.6	63.3	58.9	50.6	67.1	25
Rajshahi	27.1	18.7	37.6	89.1	47.3	98.7	78.2	45.6	93.9	20.6	17.1	24.5	17.4	12.8	23.2	46.5	41.4	51.5	35
Rangpur	60.0	34.7	80.9	96.7	87.4	99.2	77.2	46.8	92.9	44.9	23.6	68.2	22.2	19.8	24.8	60.2	46.9	73.5	33
Sylhet	50.1	30.0	70.3	93.3	80.9	97.9	86.1	69.7	94.4	30.3	19.9	43.2	40.5	22.7	61.3	60.1	53.4	66.8	18
Total	47.7	42.6	52.8	92.9	85.8	96.6	74.8	66.1	81.8	30.6	26.7	34.8	32.7	27.5	38.3	55.7	52.6	58.9	280

Note: LB and UB refer to the lower and upper bounds of the 95% confidence interval.

Table S2e. Service readiness Domain B: Equipment, Bangladesh 2014 SPA

	Sterilization equipment			Delivery bed			Examination light			Delivery pack			Suction apparatus			Manual vacuum extractor			Vacuum aspirator or D and C kit			Partograph		
	%	LB	UB	%	LB	UB	%	LB	UB	%	LB	UB	%	LB	UB	%	LB	UB	%	LB	UB	%	LB	UB
**Facility type**																								
Hospital	80.7	74.6	85.7	94.8	90.3	97.3	84.7	79.0	89.1	94.8	90.3	97.3	75.1	68.4	80.8	49.0	42.4	55.6	55.4	48.3	62.2	39.0	32.6	45.9
Health Center	40.1	31.0	50.1	69.6	59.5	78.0	49.6	40.1	59.1	69.6	59.5	78.0	39.2	29.7	49.5	17.2	11.1	25.7	18.9	13.2	26.3	20.1	13.2	29.4
Dispensary or clinic	39.3	25.4	55.2	61.8	43.0	77.6	66.0	47.6	80.6	50.0	34.3	65.7	37.3	23.4	53.6	14.7	6.6	29.4	19.4	9.5	35.6	20.4	10.2	36.7
**Managing authority**																								
Public	41.0	32.7	49.8	69.0	58.7	77.7	59.1	49.6	68.0	69.0	58.7	77.7	41.4	33.0	50.3	20.5	15.0	27.4	23.3	17.1	30.8	22.8	16.3	30.9
Private or other	87.7	80.0	92.7	89.3	80.4	94.5	88.8	79.8	94.1	89.3	80.4	94.5	73.3	63.3	81.3	40.5	31.3	50.4	49.8	39.8	59.9	34.6	26.0	44.3
**Location**																								
Urban	85.8	80.2	90.0	91.0	84.8	94.8	87.3	81.1	91.6	91.0	84.8	94.8	77.7	70.6	83.5	45.6	38.7	52.5	54.0	46.7	61.2	40.3	34.0	46.8
Rural	35.7	26.7	45.9	65.7	54.2	75.6	55.9	45.3	66.0	65.7	54.2	75.6	35.4	26.3	45.7	15.8	10.0	24.1	18.1	11.6	27.1	18.9	12.0	28.5
**Division**																								
Barisal	46.0	33.2	59.4	79.7	65.0	89.2	43.7	31.7	56.5	79.7	65.0	89.2	41.6	29.1	55.3	23.1	14.8	34.2	28.8	20.0	39.5	26.7	16.9	39.4
Chittagong	50.6	39.7	61.4	70.0	54.2	82.2	55.0	40.2	68.9	70.0	54.2	82.2	49.1	36.6	61.7	30.2	21.5	40.7	36.2	26.0	47.9	17.8	11.2	27.1
Chaka	62.8	45.7	77.3	77.3	58.6	89.2	67.9	50.0	81.8	77.3	58.6	89.2	55.1	38.8	70.3	19.0	9.2	35.2	22.1	12.2	36.7	29.6	16.6	47.0
Khulna	44.8	27.6	63.4	66.6	42.4	84.4	74.0	52.7	87.9	66.6	42.4	84.4	56.2	33.5	76.6	21.8	13.9	32.6	34.9	21.1	51.8	29.2	15.9	47.4
Rajshahi	27.6	21.2	35.1	72.0	44.3	89.3	46.8	24.0	70.9	72.0	44.3	89.3	28.2	21.1	36.5	16.4	9.7	26.4	12.5	8.5	18.1	13.6	8.6	21.0
Rangpur	47.5	24.9	71.1	69.3	41.2	87.9	93.5	81.5	97.9	69.3	41.2	87.9	49.8	26.7	73.0	41.1	28.9	54.5	46.4	24.1	70.2	35.1	23.5	48.8
Sylhet	44.3	32.9	56.4	73.2	49.3	88.5	72.2	53.9	85.2	73.2	49.3	88.5	32.4	21.5	45.7	26.1	16.1	39.5	29.4	18.7	43.1	22.1	11.8	37.7
Total	50.4	43.4	57.5	73.1	64.7	80.2	65.1	57.2	72.2	73.1	64.7	80.2	47.8	40.6	55.1	24.5	19.6	30.3	28.6	23.1	34.8	25.2	19.5	31.8

	Gloves			Newborn bag and mask			Infant scale			Blood pressure apparatus, dig or manual			Soap or hand disinfectant			*Service readiness Domain B summary score*			
	%	LB	UB	%	LB	UB	%	LB	UB	%	LB	UB	%	LB	UB	Mean	LB	UB	N
**Facility type**																			
Hospital	82.0	75.5	87.1	76.9	70.1	82.6	67.0	59.8	73.4	98.1	95.3	99.2	95.3	91.5	97.5	75.3	72.7	77.9	73
Health Center	72.0	61.6	80.5	37.8	28.7	47.8	61.9	52.2	70.8	79.6	68.9	87.2	73.0	62.3	81.6	48.7	44.3	53.1	99
Dispensary or clinic	63.6	45.6	78.5	33.4	20.0	50.0	52.1	39.4	64.4	95.2	82.9	98.8	67.0	48.2	81.7	47.7	39.7	55.7	108
**Managing authority**																			
Public	69.3	59.4	77.7	40.0	31.7	48.8	57.0	49.5	64.2	88.1	81.9	92.3	71.5	61.1	79.9	50.6	46.2	54.9	224
Private or other	79.5	69.7	86.7	71.3	61.1	79.7	69.1	58.9	77.7	99.9	99.0	100.0	96.6	90.8	98.8	73.8	69.4	78.1	56
**Location**																			
Urban	81.6	74.7	86.9	74.9	67.6	81.0	70.2	62.8	76.7	98.1	95.7	99.2	95.4	91.6	97.5	75.3	72.2	78.3	82
Rural	67.1	56.1	76.6	34.4	25.5	44.6	54.9	46.5	63.1	87.3	80.3	92.0	68.7	57.2	78.3	46.9	42.1	51.8	198
**Division**																			
Barisal	83.2	68.9	91.7	48.9	35.7	62.2	50.4	37.1	63.6	90.5	76.9	96.4	70.5	55.2	82.2	52.9	46.4	59.4	15
Chittagong	66.2	49.0	80.0	43.6	32.7	55.3	53.5	40.7	65.8	83.5	65.5	93.1	71.4	54.3	83.9	52.1	44.5	59.6	58
Chaka	59.2	42.5	74.0	47.2	31.9	63.1	49.5	35.9	63.1	89.4	79.9	94.7	75.9	56.8	88.3	55.1	46.5	63.6	97
Khulna	82.0	61.8	92.8	51.3	30.6	71.6	57.8	38.1	75.2	82.9	60.1	94.0	74.7	45.4	91.3	55.6	46.8	64.4	25
Rajshahi	63.2	35.0	84.5	28.7	20.1	39.1	77.2	64.8	86.2	99.8	98.3	100.0	70.1	40.1	89.1	47.3	41.6	52.9	35
Rangpur	98.2	93.4	99.5	62.9	37.5	82.8	89.7	74.7	96.3	100.0			94.8	84.4	98.4	70.6	64.7	76.6	33
Sylhet	96.1	91.2	98.4	44.2	28.5	61.3	51.5	32.2	70.4	92.9	73.3	98.4	83.3	53.1	95.6	55.4	48.9	61.8	18
Total	71.4	63.2	78.3	46.3	39.2	53.5	59.4	53.1	65.5	90.5	85.5	93.8	76.5	68.0	83.3	55.3	51.6	58.9	280

Note: LB and UB refer to the lower and upper bounds of the 95% confidence interval.

Table S2f. Service readiness Domain C: Medicine and commodities, Bangladesh 2014 SPA

	Injectable antibiotic			Hydro-cortisone available at the facility			Injectable uterotonic			Skin disinfectant			Magnesium sulfate			IV solution with infusion set			Chlorhexidine for cord cleaning			Antibiotic eye ointment			*Service readiness Domain C summary score*			
	**%**	**LB**	**UB**	**%**	**LB**	**UB**	**%**	**LB**	**UB**	**%**	**LB**	**UB**	**%**	**LB**	**UB**	**%**	**LB**	**UB**	**%**	**LB**	**UB**	**%**	**LB**	**UB**	**Mean**	**LB**	**UB**	**N**
**Facility type**																												
Hospital	75.1	68.6	80.7	56.7	49.5	63.5	72.7	66.2	78.4	54.9	47.8	61.8	51.7	44.7	58.7	82.7	76.8	87.4	50.0	42.9	57.2	28.3	22.2	35.3	59.0	55.0	63.0	73
Health Center	10.1	6.1	16.1	1.7	1.1	2.6	14.4	9.2	21.7	18.1	11.8	26.7	8.6	4.8	15.0	8.5	5.6	12.7	20.0	13.8	28.1	20.3	14.0	28.5	12.7	9.7	15.7	99
Dispensary or clinic	24.2	13.3	39.9	11.4	7.7	16.6	26.3	18.2	36.4	15.5	11.4	20.8	14.1	7.8	24.2	28.5	16.1	45.3	30.3	16.8	48.2	24.2	12.1	42.5	21.8	14.2	29.4	108
**Managing authority**																												
Public	22.9	16.9	30.3	9.4	7.9	11.1	24.6	19.9	30.0	17.4	13.9	21.6	13.4	9.6	18.5	24.2	17.7	32.2	26.9	19.4	36.0	22.9	15.8	32.0	20.2	16.5	24.0	224
Private or other	70.5	60.4	79.0	61.0	50.1	71.0	72.3	62.2	80.6	63.5	53.0	72.9	55.8	46.0	65.2	80.8	70.9	87.9	51.4	41.2	61.5	27.7	19.6	37.5	60.4	53.6	67.1	56
**Location**																												
Urban	70.7	63.5	77.0	55.8	48.2	63.3	68.8	61.7	75.2	58.6	51.2	65.7	51.4	44.7	58.0	80.5	73.7	85.9	50.1	42.8	57.4	28.4	22.2	35.5	58.1	53.6	62.5	82
Rural	16.7	10.3	25.7	4.8	3.1	7.4	19.9	14.8	26.2	13.4	9.6	18.4	9.8	5.8	15.9	17.0	10.2	26.8	24.2	16.1	34.8	22.0	14.2	32.4	16.0	11.6	20.3	198
**Division**																												
Barisal	21.2	15.3	28.5	12.0	7.7	18.1	21.0	13.3	31.6	20.6	14.0	29.2	5.6	3.0	10.3	24.1	17.0	32.8	15.8	10.0	23.9	10.9	5.2	21.4	16.4	12.8	20.0	15
Chittagong	33.1	24.7	42.7	24.9	17.7	33.9	29.7	21.0	40.2	30.4	21.4	41.3	17.3	11.3	25.7	31.7	23.7	41.0	35.4	22.8	50.4	21.1	12.9	32.7	28.0	21.4	34.6	58
Chaka	41.5	29.3	54.8	24.3	20.0	29.2	36.3	30.4	42.8	25.7	20.2	31.9	27.2	21.8	33.3	40.2	28.7	52.9	33.8	20.6	50.2	27.7	15.3	45.0	32.1	25.7	38.5	97
Khulna	20.8	13.8	30.2	16.1	9.3	26.4	33.2	20.0	49.7	37.7	21.0	58.0	17.5	11.3	26.0	33.2	24.5	43.3	24.8	13.3	41.5	8.0	4.0	15.3	23.9	15.7	32.2	25
Rajshahi	28.9	12.3	54.1	11.8	8.0	16.9	30.4	13.2	55.6	15.8	10.5	23.0	24.5	9.0	51.5	33.9	16.0	57.9	16.4	9.2	27.8	23.1	7.5	52.6	23.1	9.1	37.0	35
Rangpur	24.3	17.2	33.1	11.8	7.6	17.8	46.8	37.3	56.7	28.7	18.5	41.6	20.8	12.4	32.8	37.1	18.3	60.8	51.3	27.8	74.3	33.4	21.5	47.8	31.8	25.0	38.6	33
Sylhet	30.6	20.7	42.7	21.3	11.7	35.5	34.0	20.5	50.8	27.9	15.2	45.7	26.4	15.5	41.1	36.9	25.7	49.7	27.1	14.5	44.8	29.0	12.7	53.3	29.1	19.4	38.9	18
Total	32.5	27.0	38.6	19.8	17.3	22.6	34.2	29.8	39.0	26.7	23.1	30.7	22.0	18.2	26.3	35.6	29.7	42.0	31.8	25.2	39.2	23.9	17.8	31.2	28.3	24.9	31.7	280

Note: LB and UB refer to the lower and upper bounds of the 95% confidence interval.

Table S2g. Service readiness Domain D: Guidelines and staffing, Bangladesh 2014 SPA

	Facility has guidelines, observed:									*At least one provider of delivery or newborn care at facility was trained in each the following areas in the last 24 months:*																									
	**IMPAC guide-lines**			**CEmOC guidelines**			**Guidelines for management of pre-term labor**			**Training in neonatal resuscitation**			**Training in early and exclusive breastfeeding**			**Training in newborn infection management**			**Training in thermal care**			**Training in cord care**			**Training in KMC**			**Supervision**			***Service readiness Domain D summary score***			
	**%**	**LB**	**UB**	**%**	**LB**	**UB**	**%**	**LB**	**UB**	**%**	**LB**	**UB**	**%**	**LB**	**UB**	**%**	**LB**	**UB**	**%**	**LB**	**UB**	**%**	**LB**	**UB**	**%**	**LB**	**UB**	**%**	**LB**	**UB**	**Mean**	**LB**	**UB**	**N**
**Facility type**																																		
Hospital	n/a			22.7	18.0	28.3	27.6	22.2	33.7	28.2	23.1	33.9	26.1	21.1	31.7	15.6	11.5	20.7	17.7	13.7	22.6	23.4	18.7	28.9	20.2	15.7	25.7	89.7	84.1	93.5	30.4	27.0	33.8	73
Health Center				21.6	15.7	29.0	20.5	14.0	29.1	22.7	15.6	31.7	15.9	10.3	23.9	10.0	5.0	18.9	11.1	6.8	17.7	18.3	12.3	26.3	12.7	7.8	20.1	92.8	84.9	96.8	25.6	21.8	29.5	99
Dispensary				22.4	11.2	39.9	17.6	8.7	32.5	13.5	7.7	22.6	21.7	11.1	38.2	7.2	3.7	13.6	13.1	7.3	22.5	13.0	7.4	21.9	14.5	6.0	31.1	98.2	94.6	99.4	25.5	20.4	30.6	108
**Managing authority**	n/a																																	
Public				23.7	16.9	32.0	21.2	15.3	28.5	21.1	16.7	26.4	21.7	15.4	29.7	9.0	6.1	13.3	13.6	9.9	18.3	17.6	13.6	22.5	16.2	10.7	23.6	95.8	92.2	97.8	27.3	24.5	30.2	224
Private or other				16.5	10.3	25.5	21.5	14.6	30.5	18.3	11.6	27.6	17.4	10.9	26.6	15.5	9.4	24.7	13.9	8.1	22.6	17.6	11.1	26.7	12.3	7.0	20.6	87.4	79.9	92.4	24.9	19.4	30.3	56
**Location**																																		
Urban	n/a			19.9	15.4	25.3	26.2	21.1	32.1	28.7	22.8	35.4	25.7	20.0	32.3	18.4	13.6	24.4	19.7	14.9	25.4	24.0	18.8	30.2	18.4	14.3	23.5	90.2	84.8	93.8	30.6	26.9	34.3	82
Rural				23.2	15.8	32.8	19.2	12.8	27.7	17.2	12.4	23.3	18.8	12.0	28.3	7.0	3.9	12.2	11.1	7.2	16.8	14.9	10.6	20.6	14.1	8.3	23.0	95.7	91.7	97.9	25.3	22.0	28.6	198
**Division**																																		
Barisal	n/a			18.1	10.6	29.3	20.8	12.4	32.6	20.9	12.9	32.3	20.0	12.7	30.0	12.2	7.6	19.1	22.3	14.4	32.9	22.9	14.8	33.5	12.6	7.4	20.5	90.6	76.9	96.5	27.4	22.2	32.7	15
Chittagong				21.9	11.5	37.8	16.3	9.4	26.8	24.1	15.8	34.8	26.9	15.4	42.5	12.8	6.9	22.6	14.8	8.5	24.5	18.6	11.2	29.2	16.7	9.7	27.2	96.7	92.0	98.7	29.0	23.1	35.0	58
Chaka				16.7	7.5	33.0	20.3	10.1	36.8	12.9	8.5	19.0	16.9	7.7	33.1	8.1	4.0	15.7	8.5	4.8	14.7	14.6	9.6	21.6	15.2	6.4	32.0	93.2	87.7	96.4	23.3	19.0	27.5	97
Khulna				20.2	9.0	39.3	12.7	7.0	21.9	48.7	28.0	69.8	34.9	17.2	58.1	21.8	11.2	38.0	35.7	18.2	58.0	45.3	25.9	66.2	36.8	19.3	58.7	90.1	68.5	97.4	41.7	30.0	53.4	25
Rajshahi				10.1	5.4	18.1	11.9	6.6	20.6	7.4	2.6	19.2	3.0	1.3	7.0	1.4	0.4	5.4	1.9	0.6	5.3	2.6	0.9	6.8	2.6	0.9	6.8	92.9	78.3	98.0	15.2	12.8	17.7	35
Rangpur				56.2	31.2	78.5	42.4	30.4	55.3	17.6	9.3	30.8	20.5	11.2	34.6	9.3	3.4	23.3	8.2	4.1	15.8	11.2	6.0	20.0	10.3	5.3	19.3	95.3	78.4	99.1	28.6	23.3	33.9	33
Sylhet				20.5	10.1	36.9	33.8	16.5	56.8	43.5	24.1	65.0	39.5	20.9	61.8	16.8	4.5	46.2	33.1	16.3	55.9	28.9	16.5	45.7	19.4	9.8	34.6	99.5	96.5	99.9	37.7	26.5	48.8	18
Total	n/a			22.2	16.6	29.0	21.2	16.2	27.3	20.6	16.7	25.1	20.8	15.5	27.4	10.4	7.6	14.0	13.6	10.4	17.7	17.6	14.0	21.8	15.4	10.8	21.5	94.1	91.2	96.1	26.8	24.3	29.4	280

Note: LB and UB refer to the lower and upper bounds of the 95% confidence interval.

Table S3a. Service availability Domain A: BEmOC signal functions, Haiti 2013 SPA

	Parenteral admini-stration of antibiotics	Parenteral admini-stration of uterotonic drugs	Parenteral admini-stration of anti-convulsants	Manual removal of placenta	Assisted vaginal delivery	Removal of retained products	*Service availability Domain A summary score*	
	%	%	%	%	%	%	Mean	N
**Facility type**								
Hospital	90.4	98.9	71.3	70.2	94.7	72.3	83.0	94
Health Center	56.4	74.3	25.7	55.2	80.2	50.9	57.1	167
Dispensary or clinic	29.7	38.3	7.8	30.5	50.8	27.3	30.7	128
**Managing authority**								
Public	53.3	69.7	31.7	55.4	74.3	53.2	56.3	195
Private	58.2	67.0	29.9	45.9	73.7	43.3	53.0	194
**Location**								
Urban	80.2	90.7	52.2	67.6	88.7	66.9	74.4	151
Rural	40.3	54.2	17.2	39.9	64.7	36.5	42.1	238
**Department**								
Ouest	70.6	72.5	35.3	55.9	79.4	53.9	61.3	102
Sud-Est	40.0	45.7	17.1	34.3	65.7	48.6	41.9	35
Nord	61.1	66.7	36.1	47.2	80.6	50.0	56.9	36
Nord-Est	44.4	66.7	25.9	44.4	74.1	44.4	50.0	27
Artibonite	47.2	62.3	32.1	54.7	64.2	43.4	50.6	53
Centre	52.2	78.3	34.8	47.8	78.3	56.5	58.0	23
Sud	75.0	100.0	37.5	58.3	87.5	50.0	68.1	24
Grand-Anse	42.9	76.2	38.1	66.7	71.4	66.7	60.3	21
Nord-Ouest	47.1	58.9	23.3	43.3	64.5	33.3	45.1	52
Nippes	52.9	76.5	23.5	52.9	82.4	41.2	54.9	17
**Total**	**55.8**	**68.4**	**30.8**	**50.6**	**74.0**	**48.3**	**54.6**	**389**

Note: Confidence intervals are not shown since the Haiti 2013 SPA was a census of all formal health facilities, rather than a sample.

Table S3b. Service availability Domain B: Newborn signal functions, Haiti 2013 SPA

	Neonatal resuscitation	Cortisosteroids for pre-term labor	Kangaroo Mother Care	*Service availability Domain B summary score*	
	**%**	**%**	**%**	**Mean**	**N**
**Facility type**					
Hospital	66.0	51.1	27.7	48.2	94
Health Center	45.4	6.7	26.3	26.1	167
Dispensary or clinic	19.6	0.8	20.3	13.6	128
**Managing authority**					
Public	44.0	15.9	24.1	28.0	195
Private	39.7	14.9	25.3	26.6	194
**Location**					
Urban	59.5	30.5	26.4	38.8	151
Rural	30.7	5.9	23.5	20.0	238
**Department**					
Ouest	48.0	23.5	22.5	31.4	102
Sud-Est	25.7	5.7	20.0	17.1	35
Nord	38.9	19.4	25.0	27.8	36
Nord-Est	25.9	7.4	25.9	19.8	27
Artibonite	39.6	9.4	22.6	23.9	53
Centre	47.8	26.1	43.5	39.1	23
Sud	50.0	16.7	20.8	29.2	24
Grand-Anse	42.9	4.8	23.8	23.8	21
Nord-Ouest	40.9	13.8	29.2	27.9	52
Nippes	58.8	11.8	17.6	29.4	17
**Total**	**41.9**	**15.4**	**24.7**	**27.3**	**389**

Note: Confidence intervals are not shown since the Haiti 2013 SPA was a census of all formal health facilities, rather than a sample.

Table S3c. Service availability Domain C: Routine perinatal care, Haiti 2013 SPA

	Partograph routinely used to monitor labor	Early initiation of breastfeeding	Thermal care	*Service availability Domain C summary score*	
	%	%	%	Mean	N
**Facility type**					
Hospital	34.0	87.2	100.0	73.8	94
Health Center	24.5	91.6	92.8	69.7	167
Dispensary or clinic	1.6	91.4	93.8	62.2	128
**Managing authority**					
Public	21.5	92.3	95.4	69.7	195
Private	17.0	88.7	94.3	66.7	194
**Location**					
Urban	32.4	89.4	98.0	73.3	151
Rural	10.9	91.2	92.9	65.0	238
**Department**					
Ouest	22.5	86.3	97.1	68.6	102
Sud-Est	22.9	85.7	97.1	68.6	35
Nord	13.9	97.2	94.4	68.5	36
Nord-Est	11.1	85.2	85.2	60.5	27
Artibonite	20.8	88.7	88.7	66.0	53
Centre	26.1	95.7	95.7	72.5	23
Sud	25.0	100.0	100.0	75.0	24
Grand-Anse	23.8	90.5	95.2	69.8	21
Nord-Ouest	5.8	96.1	96.1	66.0	52
Nippes	29.4	88.2	100.0	72.5	17
**Total**	**19.2**	**90.5**	**94.9**	**68.2**	**389**

Note: Confidence intervals are not shown since the Haiti 2013 SPA was a census of all formal health facilities, rather than a sample.

Table S3d. Service readiness Domain A: General requirements, Haiti 2013 SPA

	Electricity	Improved water source	Improved sanitation	24/7 skilled birth attendance	Emergency transport	*Service readiness Domain A summary score*	
	%	%	%	%	%	Mean	N
**Facility type**							
Hospital	97.9	77.7	71.3	71.3	58.5	75.3	94
Health Center	87.4	79.1	55.6	38.3	29.9	58.1	167
Dispensary or clinic	53.2	67.2	18.7	2.3	13.2	30.9	128
**Managing authority**							
Public	73.8	73.3	39.9	34.2	28.6	50.0	195
Private or other	83.5	76.3	54.6	34.5	34.0	56.6	194
**Location**							
Urban	92.7	75.5	62.8	58.8	41.7	66.3	151
Rural	69.7	74.3	37.3	18.9	24.7	45.0	238
**Department**							
Ouest	83.3	72.5	78.4	38.2	41.2	62.7	102
Sud-Est	68.6	85.7	28.6	22.9	25.7	46.3	35
Nord	88.9	77.8	36.1	41.7	30.6	55.0	36
Nord-Est	70.4	66.7	37.0	33.3	14.8	44.4	27
Artibonite	64.2	64.2	43.4	35.8	43.4	50.2	53
Centre	87.0	69.6	47.8	47.8	47.8	60.0	23
Sud	95.8	87.5	45.8	33.3	37.5	60.0	24
Grand-Anse	90.5	85.7	38.1	52.4	14.3	56.2	21
Nord-Ouest	74.5	70.6	15.6	17.4	7.8	37.2	52
Nippes	70.6	94.1	58.8	29.4	35.3	57.6	17
**Total**	**78.7**	**74.8**	**47.2**	**34.4**	**31.3**	**53.3**	**389**

Note: Confidence intervals are not shown since the Haiti 2013 SPA was a census of all formal health facilities, rather than a sample.

Table S3e. Service readiness Domain B: Equipment, Haiti 2013 SPA

	Sterilization equipment	Delivery bed	Examination light	Delivery pack	Suction apparatus	Manual vacuum extractor	Vacuum aspirator or D and C kit	Partograph	Gloves	Newborn bag and mask	Infant scale	Blood pressure apparatus, dig or manual	Soap or hand disinfectant	*Service readiness Domain B summary score*	
	%	%	%	%	%	%	%	%	%	%	%	%	%	Mean	N
**Facility type**															
Hospital	89.4	97.9	52.1	94.7	54.3	28.7	35.1	40.4	92.6	75.5	90.4	92.6	79.8	71.0	94
Health Center	55.1	96.4	37.7	83.3	26.9	6.6	22.1	31.1	93.9	35.3	74.8	91.6	69.4	55.7	167
Dispensary or clinic	18.8	86.7	23.4	63.3	7.8	0.8	4.7	3.9	87.5	7.8	58.6	94.6	64.0	40.1	128
**Managing authority**															
Public	46.6	95.4	30.7	75.9	22.0	6.6	17.9	28.6	88.6	30.2	78.4	93.9	64.6	52.3	195
Private or other	56.2	91.8	42.3	83.0	32.5	13.4	21.1	20.1	94.3	41.7	68.0	91.8	75.8	56.3	194
**Location**															
Urban	70.8	98.7	45.0	90.1	40.3	16.5	31.7	39.0	90.7	56.2	86.0	92.1	70.2	63.6	151
Rural	39.0	90.3	31.1	72.7	18.9	5.9	11.7	15.1	92.0	23.1	65.1	93.3	70.1	48.3	238
**Departement**															
Ouest	56.9	93.1	47.1	80.4	45.1	16.7	29.4	23.5	93.1	50.0	72.5	93.1	79.4	60.0	102
Sud-Est	45.7	88.6	28.6	82.9	11.4	2.9	20.0	25.7	94.3	25.7	74.3	94.3	71.4	51.2	35
Nord	50.0	91.7	27.8	86.1	25.0	13.9	25.0	19.4	88.9	38.9	91.7	91.7	63.9	54.9	36
Nord-Est	63.0	92.6	37.0	77.8	14.8	11.1	14.8	25.9	96.3	18.5	66.7	81.5	66.7	51.3	27
Artibonite	37.7	92.5	26.4	64.2	24.5	5.7	13.2	26.4	92.5	28.3	62.3	92.5	64.2	48.5	53
Centre	78.3	100.0	39.1	95.7	13.0	13.0	4.3	34.8	91.3	47.8	91.3	100.0	78.3	60.5	23
Sud	62.5	100.0	50.0	91.7	45.8	4.2	25.0	29.2	87.5	58.3	79.2	91.7	70.8	61.2	24
Grand-Anse	28.6	90.5	42.9	85.7	33.3	14.3	14.3	42.9	100.0	28.6	76.2	81.0	52.4	53.1	21
Nord-Ouest	44.9	94.1	23.4	70.6	11.6	5.8	11.6	7.8	82.2	13.5	66.6	98.1	64.6	45.8	52
Nippes	52.9	100.0	47.1	82.4	17.6	0.0	17.6	35.3	94.1	47.1	64.7	100.0	76.5	56.6	17
**Total**	**51.4**	**93.6**	**36.5**	**79.4**	**27.2**	**10.0**	**19.5**	**24.4**	**91.5**	**35.9**	**73.2**	**92.8**	**70.2**	**54.3**	**389**

Note: Confidence intervals are not shown since the Haiti 2013 SPA was a census of all formal health facilities, rather than a sample.

Table S3f. Service readiness Domain C: Medicine and commodities, Haiti 2013 SPA

	Injectable antibiotic	Hydrocortisone available at the facility	Injectable uterotonic	Skin disinfectant	Magnesium sulfate	IV solution with infusion set	Chlorhexidine for cord cleaning	Antibiotic eye ointment	*Service readiness Domain C summary score*	
	%	%	%	%	%	%	%	%	Mean	N
**Facility type**										
Hospital	47.9	56.4	75.5	75.5	72.3	48.9	44.7	60.6	60.2	94
Health Center	34.7	29.9	51.4	64.0	41.9	44.2	47.9	53.3	45.9	167
Dispensary or clinic	14.0	10.9	35.2	56.2	13.3	33.6	38.2	35.1	29.6	128
**Managing authority**										
Public	25.0	22.5	50.2	61.4	40.4	40.9	45.6	45.1	41.4	195
Private or other	37.1	37.6	53.6	67.0	39.2	42.8	42.3	53.1	46.6	194
**Location**										
Urban	44.3	40.3	64.8	71.4	58.8	45.6	45.0	57.6	53.5	151
Rural	22.7	23.5	43.7	59.6	27.7	39.5	43.2	43.7	37.9	238
**Department**										
Ouest	41.2	34.3	52.9	65.7	45.1	41.2	43.1	46.1	46.2	102
Sud-Est	20.0	20.0	51.4	51.4	28.6	40.0	51.4	40.0	37.9	35
Nord	33.3	36.1	55.6	61.1	36.1	50.0	47.2	63.9	47.9	36
Nord-Est	33.3	25.9	51.9	66.7	37.0	51.9	48.1	44.4	44.9	27
Artibonite	20.8	26.4	43.4	66.0	35.8	41.5	37.7	60.4	41.5	53
Centre	43.5	56.5	56.5	78.3	65.2	52.2	43.5	60.9	57.1	23
Sud	37.5	54.2	62.5	75.0	54.2	37.5	66.7	54.2	55.2	24
Grand-Anse	28.6	23.8	61.9	66.7	47.6	42.9	47.6	42.9	45.2	21
Nord-Ouest	17.5	9.7	46.8	54.6	27.2	31.2	25.5	41.0	31.7	52
Nippes	35.3	29.4	47.1	70.6	29.4	41.2	58.8	35.3	43.4	17
**Total**	**31.1**	**30.0**	**51.9**	**64.2**	**39.8**	**41.9**	**43.9**	**49.1**	**44.0**	**389**

Note: Confidence intervals are not shown since the Haiti 2013 SPA was a census of all formal health facilities, rather than a sample.

Table S3g. Service readiness Domain D: Guidelines and staffing, Haiti 2013 SPA

	Facility has guidelines, observed:			*At least one provider of delivery or newborn care at facility was trained in each the following areas in the last 24 months:*								
	**IMPAC guidelines**	**CEmOC guidelines**	**Guidelines for management of pre-term labor**	**Training in neonatal resuscitation**	**Training in early and exclusive breastfeeding**	**Training in newborn infection management**	**Training in thermal care**	**Training in cord care**	**Training in KMC**	**Super-vision**	***Service readiness Domain D summary score***	
	**%**	**%**	**%**	**%**	**%**	**%**	**%**	**%**	**%**	**%**	**Mean**	**N**
**Facility type**												
Hospital	23.4	21.3	20.2	67.0	62.8	51.1	63.8	63.8	54.3	88.3	55.1	[illegible]
Health Center	22.2	16.7	12.6	34.2	38.3	31.2	34.7	37.7	26.3	90.4	36.8	[illegible]
Dispensary or clinic	22.6	17.2	7.8	10.9	17.1	13.2	11.7	12.5	5.4	81.2	21.3	[illegible]
**Managing authority**												
Public	24.6	15.9	11.8	30.7	33.7	28.2	33.7	34.2	25.6	89.2	35.1	[illegible]
Private or other	20.6	20.1	13.9	38.1	40.7	32.0	34.5	37.1	26.8	84.5	37.2	[illegible]
**Location**												
Urban	25.2	17.9	17.2	55.0	52.2	44.4	49.6	50.9	40.3	89.4	47.2	[illegible]
Rural	21.0	18.1	10.1	21.4	27.7	21.0	24.3	26.0	17.2	85.3	29.1	[illegible]
**Departement**												
Ouest	15.7	15.7	12.7	43.1	51.0	39.2	43.1	46.1	32.4	78.4	40.5	[illegible]
Sud-Est	31.4	14.3	5.7	20.0	28.6	22.9	31.4	31.4	20.0	94.3	32.7	[illegible]
Nord	19.4	19.4	16.7	33.3	30.6	25.0	27.8	27.8	25.0	91.7	33.3	[illegible]
Nord-Est	29.6	18.5	3.7	25.9	37.0	25.9	29.6	33.3	25.9	100.0	36.2	[illegible]
Artibonite	26.4	17.0	13.2	28.3	34.0	32.1	26.4	30.2	24.5	88.7	34.2	[illegible]
Centre	21.7	21.7	21.7	43.5	39.1	39.1	47.8	47.8	47.8	95.7	44.9	[illegible]
Sud	25.0	20.8	12.5	45.8	37.5	29.2	37.5	37.5	33.3	91.7	39.8	[illegible]
Grand-Anse	19.0	33.3	23.8	38.1	33.3	23.8	38.1	47.6	19.0	81.0	37.0	[illegible]
Nord-Ouest	25.5	13.7	9.7	21.6	19.4	17.7	17.4	13.6	5.8	80.4	23.9	[illegible]
Nippes	23.5	23.5	17.6	52.9	52.9	35.3	52.9	52.9	41.2	94.1	47.7	[illegible]
**Total**	**22.6**	**18.0**	**12.8**	**34.4**	**37.2**	**30.1**	**34.1**	**35.7**	**26.2**	**86.9**	**36.1**	[illegible]

Note: Confidence intervals are not shown since the Haiti 2013 SPA was a census of all formal health facilities, rather than a sample.

Table S4a. Service availability Domain A: BEmOC signal functions, Tanzania 2014-15 SPA

	Parenteral administration of antibiotics			Parenteral administration of uterotonic drugs			Parenteral administration of anticonvulsants			Manual removal of placenta			Assisted vaginal delivery			Removal of retained products			*Service availability Domain A summary score*			
	**%**	**LB**	**UB**	**%**	**LB**	**UB**	**%**	**LB**	**UB**	**%**	**LB**	**UB**	**%**	**LB**	**UB**	**Mean**	**LB**	**UB**	**Mean**	**LB**	**UB**	**N**
**Facility type**																						
Hospital	83.9	69.7	92.2	96.9	93.8	98.4	77.5	65.3	86.3	69.7	58.8	78.7	88.9	84.2	92.4	68.6	57.9	77.6	80.9	74.5	87.3	44
Health Center	60.0	54.5	65.3	90.9	86.8	93.9	31.6	26.7	37.0	45.3	40.0	50.8	72.9	67.7	77.4	51.4	46.2	56.5	58.7	55.7	61.6	109
Dispensary or clinic	27.3	22.8	32.4	81.8	77.3	85.5	7.0	4.6	10.4	30.2	25.5	35.5	68.0	62.9	72.7	31.1	26.8	35.8	40.9	38.5	43.2	751
**Managing authority**																						
Public	32.0	27.8	36.7	83.7	79.4	87.2	10.9	8.6	13.7	32.9	28.4	37.6	69.5	64.7	73.9	34.9	30.7	39.3	44.0	41.8	46.2	756
Private or other	44.0	33.9	54.7	83.2	73.2	89.9	25.6	18.5	34.4	39.5	30.0	49.8	70.1	59.1	79.2	37.8	28.4	48.2	50.0	44.2	55.8	149
**Location**																						
Urban	42.7	32.8	53.1	76.7	65.5	85.1	24.5	19.4	30.4	38.1	29.6	47.5	72.9	62.3	81.4	32.7	25.4	41.0	47.9	42.5	53.4	132
Rural	32.5	28.1	37.2	84.8	80.7	88.2	11.4	8.9	14.5	33.2	28.7	38.1	69.0	64.3	73.4	35.8	31.7	40.1	44.5	42.2	46.7	773
**Province**																						
Eastern	31.1	20.7	43.9	85.2	72.0	92.8	23.6	14.1	36.8	27.8	16.9	42.1	59.1	45.9	71.1	25.9	15.5	40.0	42.1	34.9	49.4	115
Western	43.9	32.1	56.5	82.2	68.0	90.9	25.9	16.6	38.0	38.9	26.7	52.6	81.9	70.3	89.7	39.7	27.1	53.9	52.1	45.1	59.1	85
Southern	26.4	15.9	40.5	91.8	78.0	97.2	5.3	3.7	7.5	59.7	44.1	73.5	75.9	60.2	86.8	34.8	22.2	49.9	49.0	41.7	56.3	66
Southern Highlands	21.7	13.8	32.4	72.4	59.6	82.4	8.1	5.0	13.0	31.2	21.1	43.6	57.6	44.6	69.7	32.7	22.7	44.5	37.3	31.9	42.7	115
SW Highlands	43.8	29.8	58.9	95.0	80.3	98.9	7.3	5.3	9.9	27.7	17.5	40.9	56.2	41.7	69.7	26.6	20.7	33.5	42.8	37.2	48.3	103
Central	50.9	38.0	63.6	90.4	78.7	96.0	13.5	8.2	21.5	36.9	25.7	49.7	78.6	67.9	86.5	64.1	51.3	75.1	55.7	51.2	60.2	104
Northern	32.5	21.5	45.9	89.6	79.6	95.0	18.8	11.3	29.7	31.9	20.0	46.9	78.3	64.6	87.7	22.4	13.4	35.0	45.6	39.3	51.9	110
Lake	28.4	20.8	37.4	73.9	63.8	82.0	7.6	4.5	12.4	31.6	23.5	41.1	74.1	64.3	82.0	37.6	29.9	45.8	42.2	38.4	45.9	199
Zanzibar	24.3	12.6	41.7	92.5	72.0	98.3	19.3	10.3	33.4	22.8	13.7	35.4	30.7	19.6	44.7	30.9	19.1	45.8	36.7	28.8	44.7	7
**Total**	**34.0**	**30.1**	**38.2**	**83.6**	**79.9**	**86.8**	**13.3**	**11.1**	**16.0**	**34.0**	**29.9**	**38.3**	**69.6**	**65.3**	**73.5**	**35.4**	**31.6**	**39.3**	**45.0**	**43.0**	**47.0**	**905**

Note: LB and UB refer to the lower and upper bounds of the 95% confidence interval.

Table S4b. Service availability Domain B: Newborn signal functions, Tanzania 2014-15 SPA

	Neonatal resuscitation			Cortisosteroids for pre-term labor			Kangaroo Mother Care			*Service availability Domain B summary score*			
	**%**	**LB**	**UB**	**%**	**LB**	**UB**	**%**	**LB**	**UB**	**Mean**	**LB**	**UB**	**N**
**Facility type**													
Hospital	92.0	87.8	94.9	43.4	35.6	51.5	65.6	55.4	74.6	67.0	61.7	72.3	44
Health Center	73.0	68.1	77.5	9.2	6.4	13.0	44.9	39.5	50.6	42.4	39.5	45.3	109
Dispensary or clinic	46.8	41.7	51.9	1.0	0.3	2.8	15.5	12.0	19.8	21.1	18.8	23.4	751
**Managing authority**													
Public	50.6	45.9	55.4	2.9	2.0	4.3	19.8	16.3	23.7	24.4	22.3	26.6	756
Private or other	59.8	48.3	70.2	9.7	7.2	12.9	30.2	22.3	39.6	33.2	27.6	38.9	149
**Location**													
Urban	58.1	47.3	68.3	12.6	9.5	16.5	29.5	22.5	37.7	33.4	27.9	38.9	132
Rural	51.1	46.3	55.9	2.6	1.7	3.9	20.1	16.6	24.1	24.6	22.4	26.8	773
**Province**													
Eastern	37.1	25.2	50.8	6.0	4.2	8.5	26.6	17.1	38.9	23.3	17.6	28.9	115
Western	50.9	36.7	65.0	8.2	3.5	18.0	14.4	8.0	24.6	24.5	17.4	31.6	85
Southern	58.5	43.1	72.4	0.9	0.3	2.7	29.5	18.0	44.4	29.6	22.7	36.5	66
Southern Highlands	74.7	61.8	84.3	2.2	1.3	3.8	9.0	5.7	13.9	28.6	24.5	32.8	115
SW Highlands	61.5	46.1	74.9	2.3	1.4	4.0	34.4	22.5	48.6	32.7	26.1	39.3	103
Central	59.3	47.2	70.3	3.1	2.1	4.5	20.5	13.3	30.2	27.6	23.0	32.3	104
Northern	56.6	43.9	68.4	3.8	2.5	5.8	34.7	22.4	49.5	31.7	25.1	38.3	110
Lake	35.7	27.4	45.0	4.4	2.3	8.4	13.0	9.1	18.2	17.7	13.5	22.0	199
Zanzibar	37.0	22.8	53.8	11.4	4.6	25.7	11.8	4.7	26.6	20.0	10.8	29.3	7
**Total**	**52.1**	**47.8**	**56.5**	**4.0**	**3.1**	**5.2**	**21.5**	**18.4**	**25.0**	**25.9**	**23.9**	**27.9**	**905**

Note: LB and UB refer to the lower and upper bounds of the 95% confidence interval.

Table S4c. Service availability Domain C: Routine perinatal care, Tanzania 2014-15 SPA

	Partograph routinely used to monitor labor			Routine early initiation of breastfeeding (w/in first hour)			Thermal care			*Service availability Domain C summary score*			
	**%**	**LB**	**UB**	**%**	**LB**	**UB**	**%**	**LB**	**UB**	**Mean**	**LB**	**UB**	**N**
**Facility type**													
Hospital	91.1	86.9	94.0	98.0	95.3	99.2	99.2	96.7	99.8	96.1	94.6	97.6	44
Health Center	69.6	64.6	74.3	97.8	95.1	99.0	97.9	95.1	99.1	88.4	86.4	90.5	109
Dispensary or clinic	38.5	33.5	43.8	98.5	96.4	99.4	97.8	95.2	99.0	78.3	76.3	80.3	751
**Managing authority**													
Public	43.6	38.8	48.6	98.3	96.3	99.2	97.6	95.1	98.8	79.8	77.9	81.7	756
Private or other	51.1	40.6	61.5	98.9	96.9	99.6	99.4	97.2	99.9	83.1	79.5	86.7	149
**Location**													
Urban	56.8	45.0	67.9	96.2	87.9	98.8	95.2	85.5	98.5	82.7	77.1	88.3	132
Rural	42.8	38.1	47.7	98.8	97.1	99.5	98.4	96.0	99.3	80.0	78.2	81.7	773
**Province**													
Eastern	50.8	36.8	64.7	96.5	85.3	99.2	94.0	81.6	98.2	80.4	73.8	87.1	115
Western	32.9	21.5	46.7	99.7	97.7	100.0	97.6	84.4	99.7	76.7	72.5	80.9	85
Southern	60.6	44.7	74.5	100.0			100.0			86.9	81.7	92.0	66
Southern Highlands	39.9	29.1	51.8	100.0			95.8	85.9	98.8	78.6	73.9	83.2	115
SW Highlands	37.5	24.6	52.5	100.0			96.4	77.6	99.5	78.0	72.3	83.6	103
Central	35.9	24.7	48.9	92.8	81.2	97.5	99.7	98.2	100.0	76.1	71.4	80.9	104
Northern	73.8	60.8	83.7	99.6	97.3	99.9	99.6	97.4	99.9	91.0	87.2	94.9	110
Lake	36.5	28.0	46.1	99.3	95.9	99.9	99.7	98.8	99.9	78.5	75.4	81.6	199
Zanzibar	48.6	35.5	62.0	86.0	70.1	94.1	96.6	78.6	99.5	77.1	69.3	84.8	7
**Total**	**44.8**	**40.5**	**49.2**	**98.4**	**96.8**	**99.2**	**97.9**	**95.8**	**99.0**	**80.4**	**78.7**	**82.0**	**905**

Note: LB and UB refer to the lower and upper bounds of the 95% confidence interval.

Table S4d. Service readiness Domain A: General requirements, Tanzania 2014-15 SPA

	**Electricity**			**Improved water source**			**Improved sanitation**			**24/7 skilled birth attendance**			**Emergency transport**			***Service readiness Domain A summary score***			
	**%**	**LB**	**UB**	**%**	**LB**	**UB**	**%**	**LB**	**UB**	**%**	**LB**	**UB**	**%**	**LB**	**UB**	**Mean**	**LB**	**UB**	**N**
**Facility type**																			
Hospital	90.5	71.5	97.3	86.7	71.1	94.5	74.7	63.2	83.6	97.7	94.9	99	93.4	89.4	96	88.6	82.4	94.8	44
Health Center	76.1	71	80.5	82.3	77.7	86.1	49.3	43.9	54.8	82.7	78.4	86.2	75.4	70.4	79.9	73.2	70.7	75.6	109
Dispensary or clinic	63.4	58.1	68.5	57.9	52.6	63	27.8	23.5	32.6	16.3	12.7	20.7	57.7	52.4	62.9	44.6	42.1	47.2	751
**Managing authority**																			
Public	62.8	57.8	67.6	57.9	53	62.6	26.6	22.7	30.7	24.7	21.2	28.4	59.4	54.5	64	46.3	44.0	48.5	756
Private or other	83.7	73.1	90.7	84.5	73.9	91.3	63.7	52.9	73.3	46.7	36.4	57.2	73.1	62	81.8	70.3	65.7	74.9	149
**Location**																			
Urban	66.2	54.9	75.9	73.3	60.8	82.9	68.3	57.6	77.3	49	39.4	58.7	57.7	45.8	68.8	62.9	56.5	69.3	132
Rural	66.3	61.3	70.9	60.4	55.5	65	26.6	22.6	31	24.7	21.2	28.7	62.3	57.4	66.9	48.0	45.7	50.4	773
**Province**																			
Eastern	57.1	42.6	70.4	66	50.5	78.7	46.3	35.2	57.7	27.8	18.9	39	48.9	34.8	63.2	49.2	41.9	56.6	115
Western	88.1	76.2	94.5	74.3	59.5	85.1	25.9	16.6	38.1	20.3	12.8	30.6	58.2	43.3	71.7	53.4	48.2	58.5	85
Southern	68.9	52.7	81.4	61.6	47.2	74.2	32	19.7	47.4	18.2	10.7	29	36.5	23.7	51.5	43.4	36.9	49.9	66
Southern Highlands	64.3	51.1	75.6	66	53.2	76.8	26.1	17.3	37.4	18.8	13.4	25.7	42.9	32.6	53.9	43.6	38.2	49.1	115
SW Highlands	56.3	40.9	70.5	41.9	28.7	56.5	19.9	11	33.3	28.5	18.6	41.1	68.5	52.5	81.1	43.0	34.9	51.2	103
Central	66.9	53	78.3	66.5	52.6	78	18.8	11.4	29.3	24.2	16.8	33.4	78.2	65.1	87.3	50.9	45.7	56.1	104
Northern	54.6	39.9	68.6	69.9	55	81.6	28.6	19.3	40.3	33.4	23.9	44.3	67.5	52.1	79.8	50.8	43.8	57.8	110
Lake	73	64.2	80.3	56.1	47.6	64.3	45.6	36.1	55.4	40.2	31.7	49.3	74.6	64.7	82.5	57.9	53.7	62.1	199
Zanzibar	87.1	70.7	95.0	88.2	73.5	95.3	92.8	79.5	97.7	22.8	12.9	37.0	44.9	32.7	57.8	67.2	62.2	72.1	7
**Total**	**66.3**	**61.7**	**70.5**	**62.2**	**57.8**	**66.5**	**32.7**	**28.9**	**36.6**	**28.3**	**25**	**31.8**	**61.6**	**57.1**	**65.9**	**50.2**	**48.1**	**52.4**	**905**

Note: LB and UB refer to the lower and upper bounds of the 95% confidence interval.

Table S4e. Service readiness Dimension B: Equipment, Tanzania 2014-15 SPA

	Sterilization equipment			Delivery Bed			Examination light			Delivery Pack			Suction apparatus			Manual vacuum extractor			Vacuum aspirator or D and C kit			Partograph		
	%	LB	UB	%	LB	UB	%	LB	UB	%	LB	UB	%	LB	UB	%	LB	UB	%	LB	UB	%	LB	UB
**Facility type**																								
Hospital	88.5	71.6	95.9	100.0			53.6	44.9	62.2	98.1	95.4	99.2	51.9	43.4	60.2	51.9	43.4	60.2	39.8	32.6	47.4	96.4	93.2	98.1
Health Center	47.9	42.8	53.1	100.0			28.0	23.4	33.1	89.6	86.1	92.3	11.6	8.4	15.7	11.6	8.4	15.7	24.4	20.3	29.1	79.7	75.0	83.6
Dispensary or clinic	12.8	9.9	16.5	98.0	95.8	99.1	10.0	7.2	13.8	85.9	81.6	89.3	1.7	0.6	4.3	1.7	0.6	4.3	3.1	1.8	5.5	52.0	46.8	57.2
**Managing authority**																								
Public	14.4	11.9	17.3	98.0	95.9	99.1	9.2	6.9	12.1	85.2	81.1	88.6	3.8	2.6	5.6	3.8	2.6	5.6	6.0	4.6	7.9	57.0	52.1	61.8
Private or other	53.1	42.2	63.8	100.0			40.6	30.7	51.3	95.6	85.5	98.8	12.8	8.7	18.6	12.8	8.7	18.6	14.8	9.8	21.6	60.0	48.7	70.3
**Location**																								
Urban	60.8	49.8	70.8	100.0			38.3	28.3	49.3	94.3	85.9	97.9	17.5	12.7	23.7	17.5	12.7	23.7	16.7	11.8	22.9	68.4	56.0	78.7
Rural	13.9	11.2	17.1	98.1	95.9	99.1	10.2	7.8	13.4	85.7	81.6	88.9	3.2	2.0	5.0	3.2	2.0	5.0	5.9	4.4	7.8	55.6	50.8	60.4
**Province**																								
Eastern	37.3	29.3	46.2	100.0			26.7	17.3	38.8	88.3	74.9	95.1	9.0	4.8	16.3	9.0	4.8	16.3	9.1	5.7	14.2	59.8	44.8	73.2
Western	30.0	20.3	42.0	100.0			7.4	2.9	17.5	88.1	76.1	94.5	4.1	2.7	6.2	4.1	2.7	6.2	12.4	6.3	22.8	60.8	47.9	72.3
Southern	11.2	5.6	21.4	89.2	74.5	95.9	10.9	4.7	23.5	85.2	70.4	93.3	2.0	1.2	3.3	2.0	1.2	3.3	2.2	1.2	4.1	77.0	61.1	87.6
Southern Highlands	13.4	8.1	21.4	98.0	87.0	99.7	6.1	3.1	11.9	92.3	81.4	97.0	1.9	1.2	2.9	1.9	1.2	2.9	5.4	2.6	10.8	62.4	51.4	72.3
SW Highlands	10.9	5.4	20.8	96.0	78.8	99.3	26.1	16.1	39.5	95.3	80.0	99.0	1.9	1.1	3.3	1.9	1.1	3.3	0.8	0.3	1.9	51.6	37.2	65.8
Central	6.8	5.3	8.8	100.0			6.5	3.4	12.2	89.5	78.1	95.3	3.9	2.7	5.7	3.9	2.7	5.7	3.9	2.6	5.8	44.3	32.5	56.6
Northern	37.3	25.9	50.2	100.0			21.4	13.8	31.6	86.5	71.2	94.3	7.3	3.5	14.9	7.3	3.5	14.9	9.9	5.2	17.9	74.3	60.0	84.7
Lake	16.3	11.0	23.4	99.4	95.5	99.9	9.3	5.0	16.7	78.9	69.4	86.1	7.7	4.2	13.9	7.7	4.2	13.9	10.7	7.2	15.7	46.1	37.1	55.4
Zanzibar	60.6	39.5	78.3	100.0			34.3	21.1	50.5	53.6	40.5	66.2	13.7	7.1	24.8	13.7	7.1	24.8	24.8	13.5	41.1	60.8	49.4	71.2
**Total**	**20.7**	**18.0**	**23.7**	**98.4**	**96.5**	**99.2**	**14.3**	**11.8**	**17.3**	**86.9**	**83.4**	**89.8**	**5.3**	**4.0**	**6.9**	**5.3**	**4.0**	**6.9**	**7.5**	**6.0**	**9.2**	**57.5**	**53.1**	**61.8**

	Gloves			Newborn bag and mask			Infant scale			Blood pressure apparatus, dig or manual			Soap or hand disinfectant			*Service readiness Domain B summary score*			
	%	LB	UB	%	LB	UB	%	LB	UB	%	LB	UB	%	LB	UB	Mean	LB	UB	N
**Facility type**																			
Hospital	99.6	97.3	99.9	97.2	94.2	98.7	92.9	69.3	98.7	95.7	92.3	97.6	94.2	90.3	96.5	83.6	79.7	87.5	44
Health Center	88.9	85.3	91.7	84.5	80.4	87.8	95.3	92.3	97.2	70.7	65.6	75.4	79.4	74.7	83.5	65.1	63.4	66.8	109
Dispensary or clinic	85.1	81.0	88.4	73.7	69.5	77.6	76.5	71.7	80.7	66.5	61.4	71.2	64.0	58.8	68.8	49.7	48.2	51.1	751
**Managing authority**																			
Public	84.8	80.9	88.0	75.4	71.5	78.9	79.0	74.4	82.9	68.3	63.5	72.7	63.8	58.9	68.5	51.1	49.7	52.4	756
Private or other	93.5	85.8	97.1	80.0	68.6	88.0	82.4	71.7	89.7	69.0	57.6	78.6	85.2	75.2	91.6	63.9	60.0	67.8	149
**Location**																			
Urban	92.1	83.2	96.5	86.0	76.7	91.9	86.3	75.0	92.9	81.0	71.2	88.1	81.9	71.8	88.9	66.9	62.9	70.9	132
Rural	85.2	81.4	88.4	74.5	70.4	78.2	78.4	73.9	82.3	66.3	61.5	70.7	64.8	60.0	69.4	50.8	49.5	52.1	773
**Division**																			
Eastern	86.5	73.5	93.7	89.2	77.0	95.3	90.3	76.9	96.3	68.9	55.5	79.7	81.4	67.6	90.1	59.2	55.3	63.0	115
Western	73.8	59.0	84.6	76.8	62.6	86.8	82.3	69.4	90.5	52.8	39.9	65.4	47.2	33.4	61.4	50.9	46.9	54.8	85
Southern	80.1	66.3	89.2	83.4	70.1	91.5	84.1	68.7	92.8	60.5	44.8	74.2	57.5	41.9	71.7	51.2	47.4	55.1	66
Southern Highlands	92.8	82.2	97.3	94.2	83.6	98.1	74.2	61.4	83.9	78.4	65.7	87.3	87.8	76.3	94.1	56.1	53.3	58.9	115
SW Highlands	83.1	75.5	88.8	76.5	63.4	85.9	80.7	66.7	89.8	91.4	79.4	96.7	76.1	60.7	86.8	55.0	51.7	58.2	103
Central	95.1	84.6	98.5	85.4	73.7	92.4	71.7	57.9	82.3	69.4	55.6	80.4	51.0	38.4	63.4	49.7	46.8	52.5	104
Northern	87.9	74.5	94.8	96.0	84.2	99.1	79.7	66.7	88.5	77.5	63.6	87.2	78.5	64.3	88.1	60.2	56.5	63.9	110
Lake	85.3	76.8	91.1	40.8	31.7	50.5	77.0	67.4	84.4	53.9	43.9	63.6	56.4	46.2	66.2	46.4	43.3	49.5	199
Zanzibar	96.6	78.6	99.5	47.0	33.9	60.6	83.2	57.3	94.8	76.9	60.5	87.9	86.3	71.3	94.1	59.7	54.4	65.0	7

Total	**86.2**	**82.9**	**89.0**	**76.2**	**72.6**	**79.4**	**79.6**	**75.5**	**83.1**	**68.4**	**64.2**	**72.4**	**67.3**	**63.0**	**71.4**	**53.2**	**51.9**	**54.4**	**905**

Note: LB and UB refer to the lower and upper bounds of the 95% confidence interval.

Table S4f. Service readiness Domain C: Medicines and commodities, Tanzania 2014-15 SPA

	Injectable antibiotic			Hydrocortisone available at the facility			Injectable uterotonic			Skin disinfectant			Magnesium sulfate			IV solution with infusion set			Chlorhexidine for cord cleaning			Antibiotic eye ointment			*Service readiness Domain C summary score*			
	**%**	**LB**	**UB**	**%**	**LB**	**UB**	**%**	**LB**	**UB**	**%**	**LB**	**UB**	**%**	**LB**	**UB**	**%**	**LB**	**UB**	**%**	**LB**	**UB**	**%**	**LB**	**UB**	**Mean**	**LB**	**UB**	**N**
**Facility type**																												
Hospital	52.7	43.9	61.3	84.0	69.8	92.2	97.2	94.2	98.7	81.2	67.8	89.8	87.3	71.3	95.0	85.8	70.7	93.8	18.3	13.7	23.9	45.7	37.8	53.8	69.0	61.7	76.3	44
Health Center	36.0	30.8	41.5	52.3	46.9	57.7	88.1	84.3	91.1	71.6	66.5	76.2	66.8	61.3	71.8	69.0	63.7	73.8	13.8	10.2	18.4	33.5	28.5	39.0	53.9	51.5	56.3	109
Dispensary or clinic	30.4	25.6	35.6	25.5	21.1	30.4	76.4	71.4	80.7	58.4	52.9	63.6	34.3	29.4	39.4	42.9	37.5	48.5	11.3	8.4	15.1	26.2	21.9	31.1	38.2	36.0	40.3	751
**Managing authority**																												
Public	32.4	27.8	37.3	23.5	19.9	27.5	78.8	74.2	82.8	59.2	54.0	64.1	39.4	34.9	44.1	44.5	39.5	49.7	11.5	8.7	15.0	24.3	20.3	28.7	39.2	37.2	41.2	756
Private or other	30.8	22.5	40.5	72.4	60.4	81.8	78.9	67.4	87.1	70.9	60.2	79.7	47.6	37.2	58.2	66.7	54.7	76.8	14.3	8.5	23.0	47.3	36.7	58.2	53.6	48.0	59.2	149
**Location**																												
Urban	36.1	26.9	46.4	54.3	42.8	65.3	84.7	73.5	91.7	66.8	54.9	76.9	59.4	47.5	70.3	60.5	49.4	70.7	13.8	8.5	21.6	40.9	30.6	52.0	52.1	46.7	57.4	132
Rural	31.4	27.0	36.3	27.7	23.6	32.1	77.8	73.2	81.8	60.1	55.1	65.0	37.6	33.1	42.3	46.0	41.0	51.1	11.6	8.8	15.2	25.9	21.8	30.4	39.8	37.7	41.8	773
**Province**																												
Eastern	26.7	15.9	41.2	52.8	39.7	65.4	73.0	59.3	83.4	60.4	45.3	73.7	49.2	35.7	62.9	46.1	32.6	60.3	9.8	4.8	18.7	30.4	19.2	44.5	43.5	37.5	49.6	115
Western	42.3	29.1	56.7	26.4	16.1	40.0	71.5	56.6	82.8	63.4	48.7	76.0	41.3	29.5	54.2	41.6	28.5	55.9	6.7	2.5	16.6	9.7	4.2	21.0	37.9	32.6	43.1	85
Southern	35.8	22.7	51.5	16.1	9.0	27.3	74.4	58.5	85.7	72.3	56.9	83.8	51.2	36.1	66.0	29.6	18.1	44.5	9.2	3.3	22.7	10.0	3.9	23.1	37.3	30.9	43.8	66
Southern Highlands	22.1	13.7	33.5	27.3	18.2	38.8	85.0	73.2	92.1	42.6	31.3	54.7	36.3	25.5	48.8	50.0	37.5	62.5	11.0	5.5	20.9	47.5	36.3	58.9	40.2	35.3	45.1	115
SW Highlands	50.0	36.6	63.4	24.6	15.2	37.4	87.5	72.1	95.0	64.9	49.2	77.9	57.2	42.2	71.0	62.7	46.9	76.2	25.4	13.9	41.7	46.2	33.4	59.5	52.3	47.0	57.6	103
Central	35.1	24.2	47.8	38.2	27.5	50.1	81.8	69.2	90.0	55.9	44.4	66.8	21.6	13.9	31.8	47.8	35.3	60.5	5.0	1.7	14.0	29.1	18.7	42.2	39.3	34.5	44.1	104
Northern	35.6	23.0	50.6	45.1	31.2	59.8	87.8	74.3	94.7	70.5	55.5	82.0	42.3	29.9	55.8	55.5	40.8	69.4	19.7	11.8	31.0	34.5	22.2	49.3	48.9	42.1	55.6	110
Lake	23.0	15.7	32.4	21.9	15.3	30.3	71.7	61.3	80.1	62.9	52.6	72.2	34.2	26.6	42.8	44.4	35.1	54.0	9.4	5.6	15.5	16.6	10.6	24.9	35.5	31.9	39.1	199
Zanzibar	22.8	12.9	36.9	28.0	15.8	44.7	92.5	73.7	98.2	64.9	41.6	82.7	71.8	53.7	84.9	85.8	67.0	94.7	8.0	2.4	23.6	6.8	2.2	19.3	47.6	41.4	53.8	7
**Total**	**32.1**	**28.0**	**36.5**	**31.5**	**27.7**	**35.6**	**78.8**	**74.7**	**82.4**	**61.1**	**56.5**	**65.5**	**40.8**	**36.6**	**45.0**	**48.2**	**43.6**	**52.8**	**12.0**	**9.4**	**15.0**	**28.0**	**24.3**	**32.1**	**41.6**	**39.7**	**43.4**	**905**

Note: LB and UB refer to the lower and upper bounds of the 95% confidence interval.

Table S4g. Service readiness Domain D: Guidelines and staffing, Tanzania 2014-15 SPA

	Facility has guidelines, observed:									*At least one provider of delivery or newborn care at facility was trained in each the following areas in the last 24 months:*																				
	**IMPAC guidelines**			**CEmOC guidelines**			**Guidelines for management of pre-term labor**			**Training in neonatal resuscitation**			**Training in early and exclusive breastfeeding**			**Training in newborn infection management**			**Training in thermal care**			**Training in cord care**			**Training in KMC**			**Supervision**		
	**%**	**LB**	**UB**	**%**	**LB**	**UB**	**%**	**LB**	**UB**	**%**	**LB**	**UB**	**%**	**LB**	**UB**	**%**	**LB**	**UB**	**%**	**LB**	**UB**	**%**	**LB**	**UB**	**%**	**LB**	**UB**	**%**	**LB**	**UB**
**Facility type**																														
Hospital	41.1	33.8	48.8	17.9	13.3	23.6	25.4	19.8	32.0	79.0	73.1	83.9	65.7	58.6	72.3	59.6	51.7	67.0	65.7	58.5	72.2	65.4	58.1	72.0	61.4	53.8	68.5	66.8	56.6	75.6
Health Center	48.5	43.2	53.7	20.0	16.0	24.6	14.9	11.3	19.4	70.5	66.1	74.6	62.5	57.3	67.4	52.9	47.6	58.1	62.5	57.6	67.2	64.4	59.5	68.9	57.7	52.5	62.6	76.9	71.9	81.2
Dispensary or clinic	24.5	20.4	29.2	6.8	4.6	9.9	10.3	7.5	13.8	47.9	43.3	52.6	45.7	40.7	50.7	34.2	29.5	39.3	42.3	37.8	47.0	42.9	38.4	47.6	36.7	31.9	41.7	77.0	72.2	81.2
**Managing authority**																														
Public	28.2	24.3	32.5	9.1	6.8	12.0	11.3	8.6	14.5	52.0	47.7	56.1	49.2	44.6	53.8	38.8	34.3	43.5	46.7	42.4	51.0	47.4	43.1	51.7	40.1	35.7	44.7	78.3	73.8	82.2
Private or other	28.2	19.3	39.2	7.9	4.5	13.3	13.0	7.8	21.0	53.2	42.4	63.7	46.2	35.7	57.1	32.1	23.3	42.4	41.9	32.0	52.5	42.4	32.5	53.0	42.0	31.9	52.9	67.3	56.6	76.5
**Location**																														
Urban	30.5	21.9	40.9	9.9	5.9	16.1	16.0	10.1	24.4	59.3	47.7	69.9	50.2	39.1	61.3	39.2	29.3	50.2	47.9	37.9	58.0	47.8	37.8	58.0	38.7	29.6	48.7	71.2	60.3	80.1
Rural	27.8	23.9	32.2	8.7	6.5	11.5	10.8	8.2	14.1	50.9	46.5	55.3	48.4	43.7	53.1	37.4	32.9	42.2	45.5	41.1	50.1	46.4	41.9	50.9	40.7	36.1	45.5	77.4	73.0	81.3
**Province**																														
Eastern	40.2	27.9	53.9	22.8	14.2	34.5	18.4	10.5	30.1	64.9	50.2	77.2	50.5	36.7	64.2	35.2	23.0	49.8	41.8	30.3	54.2	42.0	30.5	54.5	40.2	28.4	53.2	62.0	48.8	73.6
Western	41.5	28.8	55.5	14.7	7.4	26.9	8.1	3.0	20.1	79.5	66.8	88.2	74.6	60.5	85.0	66.1	52.7	77.4	76.9	64.3	86.0	76.9	64.3	86.0	59.7	46.6	71.6	81.0	67.0	89.9
Southern	32.4	21.0	46.5	4.0	1.0	14.9	13.7	6.3	27.3	46.8	34.5	59.4	35.4	23.1	49.9	31.3	19.9	45.4	43.6	31.2	57.0	43.6	31.2	57.0	34.3	22.7	48.1	82.1	67.1	91.1
Southern Highlands	27.3	18.2	38.9	9.4	4.5	18.8	30.8	20.8	43.0	74.6	62.6	83.8	67.6	54.8	78.3	43.6	32.3	55.7	59.8	46.8	71.5	63.7	50.6	75.0	55.7	43.8	66.9	65.9	53.4	76.6
SW Highlands	21.8	15.8	29.2	1.5	0.5	4.1	4.9	2.0	11.7	36.2	23.5	51.2	43.6	29.4	59.0	34.7	21.8	50.4	31.3	19.8	45.7	29.8	18.5	44.3	36.9	23.6	52.5	85.9	70.1	94.1
Central	16.1	9.2	26.6	7.4	2.7	18.7	8.4	3.4	19.2	43.7	34.7	53.1	43.3	34.3	52.8	34.7	26.3	44.2	41.2	33.4	49.5	43.5	34.5	52.9	38.7	30.5	47.7	77.9	66.1	86.5
Northern	36.8	24.6	50.9	5.9	3.9	8.9	7.8	2.9	19.6	78.1	62.7	88.4	69.9	55.0	81.5	51.7	38.0	65.2	76.2	61.0	86.7	79.1	64.3	88.9	55.2	40.5	69.0	75.8	62.6	85.4
Lake	19.7	13.4	27.9	6.0	3.0	11.6	4.6	2.3	9.1	19.9	13.8	27.8	23.6	16.7	32.2	20.7	14.1	29.3	20.8	14.4	29.2	19.5	13.3	27.7	19.7	13.2	28.3	81.5	72.9	87.8
Zanzibar	31.3	19.7	45.8	8.0	2.4	23.6	6.8	2.2	19.1	65.8	49.8	78.8	56.7	41.4	70.8	45.3	31.1	60.3	52.1	35.8	68.0	54.4	38.0	69.9	51.0	35.8	66.0	89.8	75.0	96.2
**Total**	**28.2**	**24.7**	**32.1**	**8.9**	**6.9**	**11.4**	**11.6**	**9.2**	**14.5**	**52.2**	**48.2**	**56.1**	**48.7**	**44.5**	**52.9**	**37.7**	**33.6**	**41.9**	**45.9**	**42.0**	**49.8**	**46.6**	**42.7**	**50.5**	**40.4**	**36.4**	**44.6**	**76.5**	**72.5**	**80.1**

	Service readiness Domain D summary score			
	**Mean**	**LB**	**UB**	**N**
**Facility type**				
Hospital	58.1	54.4	61.7	44
Health Center	57.3	54.1	60.5	109
Dispensary or clinic	39.8	36.9	42.6	751
**Managing authority**				
Public	43.3	40.7	45.9	756
Private or other	40.1	33.5	46.7	149
**Location**				
Urban	43.9	37.2	50.5	132
Rural	42.6	39.9	45.3	773
**Province**				
Eastern	44.4	37.2	51.6	115
Western	63.4	55.2	71.7	85
Southern	39.3	31.6	46.9	66
Southern Highlands	52.0	45.3	58.6	115
SW Highlands	35.8	26.8	44.7	103
Central	38.5	32.6	44.4	104

Northern	58.7	51.7	65.8	110
Lake	25.7	21.0	30.4	199
Zanzibar	50.5	40.7	60.3	7
**Total**	**42.8**	**40.4**	**45.2**	**905**

Note: LB and UB refer to the lower and upper bounds of the 95% confidence interval.

Table S5a. Service availability Domain A: BEmOC signal functions, Malawi 2013-14 SPA

	Parenteral administration of antibiotics	Parenteral administration of uterotonic drugs	Parenteral administration of anticonvulsants	Manual removal of placenta	Assisted vaginal delivery	Removal of retained products	*Service availability Domain A summary score*	
	%	%	%	%	%	%	%	N
**Facility type**								
Hospital	97.9	99.0	79.6	65.2	81.6	67.4	81.8	95
Health Center	78.1	98.1	43.8	39.0	45.2	33.4	56.3	414
Dispensary or clinic	77.7	89.0	22.5	22.3	33.6	16.5	43.6	19
**Managing authority**								
Public	79.7	97.8	49.3	44.0	52.2	42.0	60.8	347
Private or other	85.4	98.3	49.6	41.5	49.7	32.9	59.6	181
**Location**								
Urban	92.4	97.4	68.6	44.7	71.0	56.0	71.7	78
Rural	79.8	98.0	46.1	42.9	47.9	35.9	58.4	450
**Region**								
north	75.6	98.1	46.4	35.8	57.1	42.4	59.2	102
central	85.3	97.1	50.4	46.0	53.8	39.6	62.0	198
south	81.2	98.7	50.0	43.9	46.6	36.7	59.5	227
**Total**	**81.6**	**98.0**	**49.4**	**43.1**	**51.3**	**38.9**	**60.4**	**528**

Note: Confidence intervals are not shown since the Malawi 2013-14 SPA was a census of all formal health facilities, rather than a sample.

Table S5b. Service availability Domain B: Newborn signal functions, Malawi 2013-14 SPA

	Neonatal resuscitation	Cortisosteroids for pre-term labor	Kangaroo Mother Care	*Service availability Domain B summary score*	
	%	%	%	Mean	N
**Facility type**					
Hospital	92.8	55.2	84.6	77.5	95
Health Center	87.8	13.2	50.3	50.4	414
Dispensary or clinic	66.4	27.6	44.5	46.2	19
**Managing authority**					
Public	89.6	19.0	55.2	54.6	347
Private or other	84.7	25.5	58.3	56.1	181
**Location**					
Urban	89.8	51.2	59.7	66.9	78
Rural	87.6	16.0	55.7	53.1	450
**Region**					
north	93.2	18.3	56.0	55.8	102
central	90.7	21.1	51.4	54.4	198
south	83.2	22.7	60.6	55.5	227
**Total**	**87.9**	**21.2**	**56.3**	**55.1**	**528**

Note: Confidence intervals are not shown since the Malawi 2013-14 SPA was a census of all formal health facilities, rather than a sample.

Table S5c. Service availability Domain D: Routine perinatal care, Malawi 2013-14 SPA

	Partograph routinely used to monitor labor	Early initiation of breastfeeding	Thermal care	*Service availability Domain D summary score*	
	%	%	%	Mean	N
**Facility type**					
Hospital	98.0	98.9	100.0	99.0	95
Health Center	92.9	98.8	99.8	97.2	414
Dispensary or clinic	83.0	100.0	100.0	94.3	19
**Managing authority**					
Public	92.7	98.9	99.7	97.1	347
Private or other	95.0	98.9	100.0	98.0	181
**Location**					
Urban	98.7	98.7	100.0	99.1	78
Rural	92.6	98.9	99.8	97.1	450
**Region**					
north	97.1	100.0	100.0	99.0	102
central	91.2	98.5	99.5	96.4	198
south	93.9	98.7	100.0	97.5	227
**Total**	**93.5**	**98.9**	**99.8**	**97.4**	**528**

Note: Confidence intervals are not shown since the Malawi 2013-14 SPA was a census of all formal health facilities, rather than a sample.

Table S5d. Service readiness Domain A: General requirements, Malawi 2013-14 SPA

	Electricity	Improved water source	Improved sanitation	24/7 skilled birth attendance	Emergency transport	*Service readiness Domain A summary score*	
	%	%	%	%	%	%	N
**Facility type**							
Hospital	79.6	98.0	56.2	86.7	95.9	83.3	95
Health Center	65.2	94.1	16.9	47.2	87.8	62.2	414
Dispensary or clinic	66.7	100.0	44.5	33.1	94.5	67.7	19
**Managing authority**							
Public	67.4	92.6	18.5	49.8	89.3	63.5	347
Private or other	68.7	99.5	37.3	61.4	89.8	71.3	181
**Location**							
Urban	72.3	96.3	57.5	83.4	92.5	80.4	78
Rural	67.1	94.8	19.3	48.6	88.9	63.7	450
**Region**							
North	69.9	88.3	24.1	36.6	89.3	61.6	102
Central	76.4	95.6	28.9	53.3	88.7	68.6	198
South	59.5	97.4	21.9	61.9	90.2	66.2	227
**Total**	**67.9**	**95.0**	**24.9**	**53.8**	**89.5**	**66.2**	**528**

Note: Confidence intervals are not shown since the Malawi 2013-14 SPA was a census of all formal health facilities, rather than a sample.

Table S5e. Service readiness Domain B: Equipment, Malawi 2013-14 SPA

	Sterili-zation equipment	Delivery bed	Examinat-ion light	Delivery pack	Suction apparatus	Manual vacuum extractor	Vacuum aspirator or D and C kit	Parto-graph	Gloves	Newborn bag and mask	Infant scale	Blood pressure apparatus	Soap or hand disinfect-ant	*Service readiness Domain B summary score*	
	%	%	%	%	%	%	%	%	%	%	%	%	%	Mean	N
**Facility type**															
Hospital	76.5	99.0	62.4	97.0	87.8	81.6	55.1	94.9	100.0	95.9	97.0	91.9	87.8	86.7	95
Health Center	19.7	98.3	23.3	91.3	57.9	32.2	17.4	86.8	97.0	89.0	94.6	71.3	72.7	65.5	414
Dispensary or clinic	44.2	100.0	60.6	88.7	50.1	22.0	16.5	83.2	94.3	66.2	100.0	89.2	66.9	67.8	19
**Managing authority**															
Public	24.0	98.0	26.1	89.3	62.0	42.0	25.7	86.8	98.0	91.9	95.8	69.4	70.5	67.7	347
Private or other	43.7	99.5	42.3	97.8	64.8	38.2	21.0	90.7	96.2	84.7	94.1	87.6	84.2	72.7	181
**Location**															
Urban	63.6	100.0	51.3	97.5	87.3	59.8	44.9	94.9	100.0	86.0	90.1	91.3	84.8	80.9	78
Rural	25.1	98.3	28.3	91.3	58.8	37.4	20.5	86.9	97.0	90.0	96.1	72.9	73.5	67.4	450
**Region**															
North	20.1	98.0	42.5	86.4	64.9	34.7	18.2	86.3	100.0	93.2	95.1	76.6	76.6	68.7	102
Central	33.7	99.0	26.5	90.7	65.2	44.5	24.9	87.2	96.6	91.6	94.6	73.5	76.0	69.5	198
South	33.0	98.3	31.3	96.1	60.2	40.1	26.0	89.7	97.0	85.8	95.8	77.0	74.0	69.6	227
**Total**	**30.8**	**98.5**	**31.7**	**92.2**	**63.0**	**40.7**	**24.1**	**88.1**	**97.4**	**89.4**	**95.2**	**75.6**	**75.2**	**69.4**	**528**

Note: Confidence intervals are not shown since the Malawi 2013-14 SPA was a census of all formal health facilities, rather than a sample.

Table S5f. Service readiness Domain C: Medicine and commodities, Malawi 2013-14 SPA

	Injectable antibiotic	Hydrocortisone available at the facility	Injectable uterotonic	Skin disinfectant	Magnesium sulfate	IV solution with infusion set	Chlorhexidine for cord cleaning	Antibiotic eye ointment	*Service readiness Domain C summary score*	
	%	%	%	%	%	%	%	%	Mean	N
**Facility type**										
Hospital	76.6	48.1	94.9	74.5	90.8	65.4	51	91.9	74.1	95
Health Center	50.6	4.5	95.3	51.1	85.6	66.9	32.2	93.7	60	414
Dispensary or clinic	55.8	27.6	94.7	49.8	28	78.1	39.1	94.3	58.4	19
**Managing authority**									0	
Public	52.5	5.6	94.9	48.6	87.6	69.1	31.4	94.1	60.5	347
Private or other	61.1	27.6	95.7	67.9	78.7	62.9	44.3	91.9	66.3	181
**Location**									0	
Urban	75	36.3	95	61.1	84.8	68.9	42.5	91.3	69.4	78
Rural	52.1	9.1	95.2	54.2	84.5	66.7	34.7	93.7	61.3	450
**Region**									0	
North	57.2	13.4	90.3	63	83.5	69	34.8	98.1	63.7	102
Central	53.4	11.2	97.1	48	83.3	64.8	30.9	90.7	59.9	198
South	56.5	14.7	95.7	58.1	86.1	68	40.6	93.6	64.2	227
**Total**	**55.5**	**13.1**	**95.2**	**55.2**	**84.5**	**67**	**35.8**	**93.4**	**62.5**	**528**

Note: Confidence intervals are not shown since the Malawi 2013-14 SPA was a census of all formal health facilities, rather than a sample.

Table S5g. Service readiness Domain D: Guidelines and staffing, Malawi 2013-14 SPA

	Facility has guidelines, observed:			*At least one provider of delivery or newborn care at facility was trained in each the following areas in the last 24 months:*								
	IMPAC guidelines	CEmOC guidelines	Guidelines for management of pre-term labor	Training in neonatal resuscitatio n	Training in early and exclusive breast-feeding	Training in newborn infection managemen t	Training in thermal care	Training in cord care	Training in KMC	Supervision	*Service readiness Domain D summary score*	N
	%	%	%	%	%	%	%	%	%	%	Mean	
**Facility type**												
Hospital	52	39.8	55.1	80.5	66.3	62.2	77.5	75.5	57.1	91.8	67.0	95
Health Center	43.1	24.8	39.3	59.1	44	36.3	49.9	51.3	37	91.7	48.6	414
Dispensary or clinic	44.6	22.3	27.8	38.6	44.5	16.7	27.6	33.1	27.6	72.2	36.4	19
**Managing authority**												
Public	43	30.1	40.4	65.2	52.5	43.5	56.7	58.4	45.5	90.1	53.9	347
Private or other	48.1	22.2	44.2	56.6	39.5	33.9	49	48.5	30.3	92.8	46.8	181
**Location**											0.0 0.0	
Urban	49.8	39.8	47.3	74.8	60	53.7	64.9	64.9	53.6	91	61.4	78
Rural	43.9	25.2	40.8	60	46	37.9	52.2	53.3	37.9	91.1	49.7	450
**Region**												
North	48.6	40.8	40.8	70.7	56.2	46.5	54.2	55.2	42.6	87.3	55.8	102
Central	41.6	23	42.1	58.8	45.1	41.1	53.4	55.4	43.6	91.6	50.4	198
South	45.7	25.2	41.9	61.4	47	36.7	54.6	54.6	36.3	92.3	50.4	227
**Total**	**44.7**	**27.4**	**41.7**	**62.2**	**48.1**	**40.2**	**54.1**	**55**	**40.2**	**91.1**	**51.4**	**528**

Note: Confidence intervals are not shown since the Malawi 2013-14 SPA was a census of all formal health facilities, rather than a sample.

Table S6a. Service availability Domain A: BEmOC signal functions, Senegal 2014 SPA

	Parenteral administration of antibiotics			Parenteral administration of uterotonic drugs			Parenteral administration of anticonvulsants			Manual removal of placenta			Assisted vaginal delivery			Removal of retained products			*Service availability Domain A summary score*			
	**%**	**LB**	**UB**	**%**	**LB**	**UB**	**%**	**LB**	**UB**	**%**	**LB**	**UB**	**%**	**LB**	**UB**	**Mean**	**LB**	**UB**	**Mean**	**LB**	**UB**	**N**
**Facility type**																						
Hospital	97.9	86.7	99.7	97.9	86.7	99.7	69.7	48.9	84.7	74.6	53.9	88.1	100.0			97.9	86.7	99.7	89.7	84.5	94.9	11
Health Center	95.8	84.0	99.0	100.0			62.2	44.6	77.0	73.1	58.7	83.8	100.0			96.6	79.6	99.5	87.9	83.7	92.2	20
Clinic	68.0	61.5	73.9	94.1	90.1	96.5	26.4	20.7	33.0	61.0	54.7	67.0	99.3	96.7	99.9	80.9	75.3	85.5	71.6	69.0	74.2	248
**Managing Authority**																						
Public	71.1	64.8	76.7	94.7	91.0	96.9	31.7	26.0	37.9	64.0	58.0	69.6	99.3	96.8	99.9	84.0	78.6	88.2	74.1	71.7	76.6	251
Private or other	72.5	52.5	86.3	94.4	74.2	99.0	22.7	10.6	42.1	48.2	30.1	66.8	100.0			71.8	51.6	85.9	68.3	60.2	76.4	29
**Locality**																						
Urban	87.4	75.9	93.8	97.8	88.7	99.6	47.9	36.5	59.6	65.5	53.4	75.8	100.0			92.4	81.8	97.0	81.8	77.8	85.8	72
Rural	65.6	58.3	72.2	93.6	89.1	96.3	24.7	18.8	31.9	61.3	54.4	67.8	99.2	96.1	99.8	79.4	73.3	84.4	70.6	67.8	73.5	207
**Region**																						
North	61.9	47.4	74.6	95.9	84.4	99.0	30.3	19.2	44.3	91.3	80.4	96.4	100.0			97.9	86.1	99.7	79.5	75.2	83.8	63
Dakar	93.3	72.1	98.7	100.0			55.0	35.6	73.0	72.0	51.2	86.3	100.0			90.1	67.5	97.6	85.1	78.2	92.0	28
Thies	84.7	65.2	94.2	96.2	76.4	99.5	46.0	28.4	64.6	62.5	43.0	78.7	100.0			84.4	64.6	94.1	79.0	73.1	84.8	35
Central	74.8	62.8	83.9	91.5	82.4	96.2	19.6	12.3	29.8	58.0	45.7	69.5	98.3	88.9	99.8	84.1	73.0	91.1	71.1	66.3	75.9	79
East	68.8	50.7	82.6	93.5	85.0	97.3	30.8	16.0	51.0	51.1	32.2	69.7	98.6	90.3	99.8	83.7	64.3	93.6	71.1	63.1	79.0	29
South	55.8	41.9	68.8	94.8	81.4	98.7	24.0	13.7	38.7	31.1	20.5	44.1	100.0			53.1	38.0	67.6	59.8	53.8	65.9	46
**Total**	**71.2**	**65.4**	**76.5**	**94.7**	**91.1**	**96.9**	**30.8**	**25.4**	**36.6**	**62.4**	**56.7**	**67.8**	**99.4**	**97.1**	**99.9**	**82.7**	**77.7**	**86.8**	**73.5**	**71.0**	**76.1**	**279**

Note: LB and UB refer to the lower and upper bounds of the 95% confidence interval.

Table S6b. Service availability Domain B: Newborn signal functions, Senegal 2014 SPA

	Neonatal resuscitation			Cortisosteroids for pre-term labor			Kangaroo Mother Care			*Service availability Domain B summary score*			
	**%**	**LB**	**UB**	**%**	**LB**	**UB**	**%**	**LB**	**UB**	**Mean**	**LB**	**UB**	**N**
**Facility type**													
Hospital	82.6	61.8	93.3	87.5	68.9	95.6	63.7	42.3	80.7	77.9	67.0	88.8	11
Health Center	90.4	76.0	96.5	41.5	28.8	55.5	54.7	41.1	67.6	62.2	55.0	69.4	20
Clinic	56.6	49.8	63.2	9.1	5.9	13.8	53.7	46.8	60.4	39.8	36.0	43.6	248
**Managing Authority**													
Public	61.2	54.8	67.3	12.9	9.6	17.3	56.0	49.4	62.4	43.4	39.8	47.0	251
Private or other	50.5	31.7	69.1	29.2	15.7	47.8	37.8	21.7	57.1	39.2	26.5	51.9	29
**Locality**													
Urban	71.2	58.8	81.1	38.8	29.0	49.6	50.9	39.1	62.6	53.6	45.8	61.5	72
Rural	56.3	48.9	63.4	6.2	3.4	10.9	55.3	47.8	62.6	39.2	35.3	43.2	207
**Region**													
North	44.2	31.1	58.1	1.8	0.6	5.8	64.9	51.9	76.1	37.0	31.1	42.9	63
Dakar	80.0	58.9	91.8	48.0	30.7	65.8	46.8	28.5	66.0	58.3	44.3	72.2	28
Thies	86.7	67.6	95.3	29.3	16.3	46.9	47.7	30.0	66.0	54.6	44.8	64.3	35
Central	61.6	49.2	72.8	7.7	4.0	14.3	59.2	47.2	70.2	42.9	36.0	49.7	79
East	60.5	42.4	76.2	18.1	7.4	37.9	49.1	30.5	68.0	42.6	32.4	52.8	29
South	46.8	31.8	62.4	10.2	4.6	21.2	43.2	28.8	58.8	33.4	24.7	42.1	46
**Total**	**60.1**	**54.0**	**66.0**	**14.6**	**11.3**	**18.7**	**54.2**	**47.9**	**60.3**	**43.0**	**39.5**	**46.4**	**279**

Note: LB and UB refer to the lower and upper bounds of the 95% confidence interval.

Table S6c. Service availability Domain C: Routine perinatal care, Senegal 2014 SPA

	Partograph routinely used to monitor labor			Routine early initiation of breastfeeding (w/in first hour)			Thermal care			*Service availability Domain C summary score*			
	%	LB	UB	%	LB	UB	%	LB	UB	Mean	LB	UB	N
**Facility type**													
Hospital	85.1	66.1	94.4	95.7	74.1	99.4	100.0			93.6	87.2	100.0	11
Health Center	93.3	80.5	98.0	92.9	79.0	97.8	100.0			95.4	91.7	99.2	20
Clinic	66.0	59.5	72.0	98.6	95.5	99.6	100.0			88.2	86.1	90.3	248
**Managing Authority**													
Public	68.7	62.6	74.2	98.2	95.4	99.3	100.0			89.0	87.0	91.0	251
Private or other	69.2	49.2	83.9	96.8	87.4	99.2	100.0			88.7	82.4	94.9	29
**Locality**													
Urban	78.5	66.7	87.0	93.5	84.4	97.4	100.0			90.7	86.9	94.4	72
Rural	65.3	58.2	71.8	99.7	97.8	100.0	100.0			88.3	86.1	90.6	207
**Region**													
North	59.6	46.9	71.1	97.8	85.7	99.7	100.0			85.8	81.7	89.9	63
Dakar	84.9	65.1	94.5	91.6	72.2	97.9	100.0			92.2	86.3	98.0	28
Thies	76.7	56.7	89.2	98.8	91.9	99.8	100.0			91.8	86.3	97.4	35
Central	71.2	59.8	80.4	98.4	93.8	99.6	100.0			89.9	86.3	93.4	79
East	76.4	55.4	89.4	100.0			100.0			92.1	86.4	97.9	29
South	56.4	41.5	70.2	100.0			100.0			85.5	80.5	90.4	46
**Total**	**68.8**	**62.9**	**74.1**	**98.1**	**95.6**	**99.2**	**100.0**			**88.9**	**87.0**	**90.9**	**279**

Note: LB and UB refer to the lower and upper bounds of the 95% confidence interval.

Table S6d. Service readiness Domain A: General requirements, Senegal 2014 SPA

	Electricity			Improved water source			Improved sanitation			24/7 skilled birth attendance			Emergency transport			*Service readiness Domain A summary score*			
	**%**	**LB**	**UB**	**%**	**LB**	**UB**	**%**	**LB**	**UB**	**%**	**LB**	**UB**	**%**	**LB**	**UB**	**Mean**	**LB**	**UB**	**N**
**Facility type**																			
Hospital	97.9	86.7	99.7	100.0			100.0			70.5	50.8	84.6	89.5	68.3	97.1	91.6	87.3	95.9	11
Health Center	74.0	57.9	85.4	100.0			100.0			68.6	52.4	81.3	92.8	79.2	97.7	87.1	83.2	90.9	20
Clinic	51.6	45.0	58.0	90.9	86.7	93.9	93.5	89.8	95.9	8.8	5.7	13.4	53.9	46.9	60.7	59.7	57.4	62.0	248
**Managing authority**																			
Public	55.1	48.7	61.4	91.4	87.2	94.3	94.1	90.5	96.5	14.6	11.1	19.0	57.1	50.4	63.6	62.5	60.3	64.7	251
Private or other	54.3	34.9	72.4	96.7	79.9	99.6	94.8	79.0	98.9	24.7	12.5	42.9	67.0	45.9	82.9	67.5	59.5	75.5	29
**Locality**																			
Urban	58.9	47.3	69.5	100.0			94.5	84.0	98.3	44.5	33.6	56.0	67.8	55.2	78.2	73.1	68.8	77.5	72
Rural	53.7	46.5	60.8	89.1	84.1	92.7	94.1	90.4	96.4	5.6	3.1	10.0	54.8	47.2	62.1	59.5	57.0	62.0	207
**Region**																			
North	39.4	27.1	53.2	96.1	86.0	99.0	100.0			6.9	3.2	14.4	55.4	41.8	68.2	59.6	55.9	63.2	63
Dakar	56.2	38.6	72.3	100.0			94.9	70.0	99.3	58.1	38.4	75.6	79.8	58.5	91.7	77.8	71.1	84.5	28
Thies	64.0	44.5	79.8	100.0			100.0			18.9	8.9	35.7	41.9	25.8	60.0	65.0	58.3	71.7	35
Central	40.5	29.6	52.4	97.6	89.9	99.4	94.9	87.5	98.0	15.2	9.2	24.0	57.3	45.0	68.8	61.1	57.1	65.0	79
East	73.9	52.9	87.7	81.9	60.6	93.0	85.2	69.7	93.5	4.4	1.3	14.1	63.6	43.4	79.9	61.8	54.9	68.7	29
South	82.4	68.7	90.9	71.6	56.6	83.0	85.8	72.2	93.4	7.2	2.9	16.7	59.2	43.5	73.2	61.3	55.5	67.0	46
**Total**	**55.1**	**49.1**	**60.9**	**91.9**	**88.2**	**94.6**	**94.2**	**90.9**	**96.4**	**15.7**	**12.3**	**19.7**	**58.1**	**51.8**	**64.2**	**63.0**	**60.9**	**65.1**	**279**

Note: LB and UB refer to the lower and upper bounds of the 95% confidence interval.

Table S6e. Service readiness Domain B: Equipment, Senegal 2014 SPA

	Sterilization equipment			Delivery bed			Examination light			Delivery pack			Suction apparatus			Manual vacuum extractor			Vacuum aspirator or D and C kit			Partograph		
	%	LB	UB	%	LB	UB	%	LB	UB	%	LB	UB	%	LB	UB	%	LB	UB	%	LB	UB	%	LB	UB
**Facility type**																								
Hospital	57.4	36.1	76.3	100.0			75.3	56.0	88.0	100.0			77.8	58.7	89.6	26.1	11.9	48.1	39.4	23.4	58.1	89.4	70.5	96.7
Health Center	9.9	4.6	19.9	100.0			73.9	59.8	84.3	100.0			71.6	55.8	83.4	16.2	8.3	29.3	33.0	22.1	46.1	97.9	86.3	99.7
Dispensary or clinic	3.6	1.7	7.4	100.0			55.0	47.9	61.8	100.0			26.5	20.9	33.0	1.1	0.3	4.5	45.2	38.6	52.0	80.9	75.0	85.7
**Managing authority**																								
Public	41.0	32.7	49.8	69.0	58.7	77.7	59.1	49.6	68.0	100.0			29.4	24.0	35.3	2.7	1.5	4.7	44.3	38.0	50.7	83.4	77.9	87.7
Private or other	87.7	80.0	92.7	89.3	80.4	94.5	88.8	79.8	94.1	100.0			53.5	34.4	71.7	8.2	2.2	26.5	42.8	25.3	62.3	74.6	54.3	87.9
**Location**										100.0														
Urban	85.8	80.2	90.0	91.0	84.8	94.8	87.3	81.1	91.6	100.0			53.9	42.0	65.3	10.0	5.6	17.2	39.9	29.0	51.9	86.2	75.1	92.9
Rural	35.7	26.7	45.9	65.7	54.2	75.6	55.9	45.3	66.0	100.0			24.1	18.3	31.2	0.9	0.2	4.0	45.6	38.5	52.8	81.2	74.6	86.3
**Division**										100.0														
North	46.0	33.2	59.4	79.7	65.0	89.2	43.7	31.7	56.5	100.0			27.5	17.2	41.0	4.7	1.6	12.8	52.0	38.1	65.6	76.8	63.5	86.4
Dakar	50.6	39.7	61.4	70.0	54.2	82.2	55.0	40.2	68.9	100.0			64.7	44.6	80.7	6.2	2.4	15.3	36.8	20.4	57.0	91.8	73.5	97.8
Thies	62.8	45.7	77.3	77.3	58.6	89.2	67.9	50.0	81.8	100.0			48.3	30.3	66.8	5.3	1.0	23.2	57.8	39.1	74.5	88.2	68.6	96.2
Central	44.8	27.6	63.4	66.6	42.4	84.4	74.0	52.7	87.9	100.0			32.2	22.2	44.2	3.1	1.5	6.5	34.3	24.5	45.6	88.8	78.8	94.4
East	27.6	21.2	35.1	72.0	44.3	89.3	46.8	24.0	70.9	100.0			18.1	8.4	34.8	0.0			19.2	7.8	40.3	84.4	63.2	94.5
South	47.5	24.9	71.1	69.3	41.2	87.9	93.5	81.5	97.9	100.0			13.0	6.5	24.2	0.0			59.5	44.1	73.2	68.1	52.9	80.3
**Total**	**50.4**	**43.4**	**57.5**	**73.1**	**64.7**	**80.2**	**65.1**	**57.2**	**72.2**	**100.0**			**31.8**	**26.6**	**37.6**	**3.2**	**1.9**	**5.6**	**44.1**	**38.1**	**50.3**	**82.5**	**77.2**	**86.7**

	Gloves			Newborn bag and mask			Infant scale			Blood pressure apparatus, dig or manual			Soap or hand disinfectant			*Service readiness Domain B summary score*			
	%	LB	UB	%	LB	UB	%	LB	UB	%	LB	UB	%	LB	UB	Mean	LB	UB	N
**Facility type**																			
Hospital	100.0			81.4	66.2	90.7	97.9	86.7	99.7	100.0			100.0			80.4	75.9	84.8	11
Health Center	100.0			69.6	53.5	82.0	97.7	85.0	99.7	87.7	72.0	95.2	100.0			73.7	70.8	76.5	20
Dispensary or clinic	95.6	91.7	97.7	44.8	38.0	51.7	91.4	86.6	94.6	81.1	76.4	85.0	95.4	91.4	97.5	63.1	61.6	64.7	248
**Managing authority**																			
Public	96.7	93.1	98.4	45.6	39.1	52.1	92.5	88.0	95.3	82.2	77.5	86.1	95.9	92.1	97.9	64.2	62.7	65.6	251
Private or other	91.1	70.4	97.8	70.0	50.5	84.2	89.2	67.9	97.0	83.5	62.8	93.8	95.9	76.0	99.4	68.2	63.0	73.4	29
**Location**																			
Urban	98.1	87.6	99.7	62.4	50.4	73.0	97.3	89.9	99.3	85.7	74.6	92.4	100.0			70.3	67.3	73.3	72
Rural	95.4	90.9	97.7	43.1	35.8	50.6	90.3	84.8	94.0	81.2	75.7	85.6	94.4	89.7	97.1	62.6	60.9	64.2	207
**Division**																			
North	87.7	74.9	94.4	48.2	34.7	62.0	85.8	73.0	93.1	47.0	34.5	59.8	89.8	77.6	95.8	59.2	56.0	62.3	63
Dakar	100.0			60.0	40.3	76.9	98.3	88.8	99.8	93.3	72.1	98.7	100.0			72.9	69.2	76.6	28
Thies	100.0			61.2	42.1	77.4	92.0	72.5	98.1	92.3	73.4	98.1	100.0			70.5	66.1	74.8	35
Central	96.7	87.3	99.2	40.5	29.7	52.4	95.1	85.7	98.4	85.7	77.1	91.4	96.7	87.3	99.2	64.3	61.4	67.1	79
East	98.3	88.6	99.8	24.4	11.9	43.3	88.8	67.3	96.8	100.0			96.6	87.7	99.1	60.0	57.4	62.6	29
South	100.0			58.1	43.0	71.9	94.2	84.0	98.1	100.0			96.8	79.7	99.6	65.9	62.4	69.3	46
**Total**	**96.1**	**92.6**	**98.0**	**48.1**	**41.9**	**54.2**	**92.1**	**87.9**	**95.0**	**82.3**	**78.1**	**85.9**	**95.9**	**92.3**	**97.8**	**64.6**	**63.2**	**66.0**	**279**

Note: LB and UB refer to the lower and upper bounds of the 95% confidence interval.

Table S6f. Service readiness Domain C: Medicine and commodities, Senegal 2014 SPA

	Injectable antibiotic			Hydrocortisone available at the facility			Injectable uterotonic			Skin disinfectant			Magnesium sulfate			IV solution with infusion set			Chlorhexidine for cord cleaning			Antibiotic eye ointment			*Service readiness Domain C summary score*			
	**%**	**LB**	**UB**	**%**	**LB**	**UB**	**%**	**LB**	**UB**	**%**	**LB**	**UB**	**%**	**LB**	**UB**	**%**	**LB**	**UB**	**%**	**LB**	**UB**	**%**	**LB**	**UB**	**Mean**	**LB**	**UB**	**N**
**Facility type**																												
Hospital	66.9	47.5	81.9	57.8	36.0	76.9	82.7	60.3	93.8	95.9	76.2	99.4	84.7	63.2	94.7	86.9	65.9	95.8	61.2	40.2	78.6	46.5	30.7	63.1	72.8	63.5	82.2	11
Health Center	64.2	49.8	76.4	66.1	50.8	78.5	81.9	66.7	91.1	90.7	76.4	96.7	55.5	40.4	69.7	74.8	60.8	85.0	52.5	40.2	64.6	54.8	43.0	66.1	67.6	60.7	74.4	20
Clinic	51.8	46.6	56.9	56.1	49.0	62.8	77.2	71.9	81.7	93.5	89.2	96.2	31.4	25.3	38.1	68.3	61.8	74.1	48.4	43.7	53.2	52.6	47.9	57.3	59.9	57.3	62.5	248
**Managing Authority**																												
Public	50.4	45.4	55.4	58.0	51.3	64.5	76.9	71.6	81.5	92.7	88.5	95.4	33.0	27.2	39.3	68.4	62.1	74.1	48.6	43.8	53.4	51.5	46.9	56.1	59.9	57.3	62.6	251
Private or other	78.5	59.1	90.2	46.6	28.3	66.0	84.8	65.1	94.3	100.0			55.2	35.8	73.1	79.1	56.3	91.8	55.0	35.7	73.0	61.5	42.1	77.8	70.1	61.4	78.8	29
**Locality**																												
Urban	57.9	46.0	68.8	50.7	39.1	62.2	77.3	65.7	85.9	86.1	75.3	92.6	45.9	34.8	57.5	75.1	63.5	84.0	49.2	37.8	60.7	51.3	39.9	62.5	61.7	55.4	67.9	72
Rural	51.7	46.1	57.3	59.0	51.4	66.2	77.9	71.9	82.9	96.0	91.9	98.1	31.5	25.1	38.8	67.5	60.3	74.0	49.3	44.1	54.4	53.0	47.8	58.0	60.7	57.9	63.6	207
**Zone**																												
North	9.4	4.1	20.2	63.6	49.1	76.1	57.6	43.2	70.8	94.5	84.2	98.2	28.7	17.7	43.1	53.2	39.3	66.7	10.2	4.4	22.1	2.0	0.3	12.9	39.9	34.3	45.4	63
Dakar	63.2	42.8	79.7	43.6	26.4	62.5	79.8	58.6	91.6	79.6	57.7	91.8	45.8	28.9	63.8	76.4	56.1	89.2	59.9	40.0	77.0	55.1	35.7	73.1	62.9	53.4	72.5	28
Thies	60.5	41.2	76.9	75.9	56.6	88.4	85.8	67.5	94.6	91.2	73.2	97.5	34.5	19.4	53.6	79.3	59.8	90.8	55.0	36.2	72.5	47.6	29.8	66.1	66.2	56.9	75.6	35
Central	55.2	46.5	63.5	48.8	36.9	60.9	74.3	68.7	79.2	94.3	86.3	97.8	33.7	24.3	44.5	64.0	53.4	73.5	44.8	37.2	52.7	61.3	54.5	67.6	59.6	56.4	62.8	79
East	91.8	83.3	96.1	59.8	39.6	77.2	93.4	73.0	98.7	100.0			34.8	19.5	54.2	80.5	59.5	92.0	84.5	75.5	90.6	79.6	58.9	91.4	78.1	71.7	84.4	29
South	75.3	61.4	85.5	52.7	37.6	67.4	94.3	81.2	98.4	96.6	85.9	99.3	41.4	27.9	56.4	82.8	68.3	91.5	78.0	70.3	84.2	92.9	79.2	97.8	76.8	71.8	81.7	46
**Total**	**53.3**	**48.6**	**58.0**	**56.8**	**50.5**	**63.0**	**77.7**	**72.9**	**81.9**	**93.4**	**89.6**	**95.9**	**35.3**	**29.7**	**41.3**	**69.5**	**63.6**	**74.8**	**49.3**	**44.9**	**53.7**	**52.5**	**48.2**	**56.8**	**61.0**	**58.6**	**63.4**	**279**

Note: LB and UB refer to the lower and upper bounds of the 95% confidence interval.

Table S6g. Service readiness Domain D: Guidelines and staffing, Senegal 2014 SPA

	Facility has guidelines, observed:									*At least one provider of delivery or newborn care at facility was trained in each the following areas in the last 24 months:*																								
	**IMPAC guidelines**			**CEmOC guidelines**			**Guidelines for management of pre-term labor**			**Training in neonatal resuscitation**			**Training in early and exclusive breastfeeding**			**Training in newborn infection management**			**Training in thermal care**			**Training in cord care**			**Training in KMC**			***Supervision***			***Service readiness Domain D summary score***			
	**%**	**LB**	**UB**	**%**	**LB**	**UB**	**%**	**LB**	**UB**	**%**	**LB**	**UB**	**%**	**LB**	**UB**	**%**	**LB**	**UB**	**%**	**LB**	**UB**	**%**	**LB**	**UB**	**%**	**LB**	**UB**	**%**	**LB**	**UB**	**Mean**	**LB**	**UB**	**N**
**Facility type**																																		
Hospital	45.5	26.1	66.4	48.1	28.3	68.5	35.5	18.1	57.9	54.3	33.5	73.6	48.1	28.5	68.3	40.1	21.8	61.6	44.3	25.3	65.3	44.3	25.3	65.3	46.9	27.6	67.2	26.3	13.2	45.6	43.4	31.7	55.0	11
Health Center	66.7	49.8	80.3	64.7	48.1	78.3	22.7	12.8	37.0	49.2	36.0	62.5	43.7	31.3	56.9	32.8	21.1	47.2	47.0	34.4	60.0	45.0	32.8	57.8	42.9	31.2	55.5	41.7	30.4	54.1	45.6	38.4	52.9	20
Clinic	66.2	59.4	72.4	56.5	49.7	63.0	9.6	6.2	14.7	30.1	24.3	36.7	34.2	28.1	41.0	26.5	21.0	32.9	32.6	26.5	39.4	34.6	28.5	41.2	28.5	22.9	34.9	50.4	43.7	57.1	36.9	33.1	40.8	248
**Managing Authority**																																		
Public	68.4	61.9	74.2	59.5	53.0	65.7	11.5	8.0	16.2	31.9	26.3	38.2	34.5	28.5	40.9	26.8	21.5	32.9	33.8	27.9	40.2	35.1	29.3	41.4	30.7	25.3	36.7	49.8	43.4	56.2	38.2	34.6	41.8	251
Private or other	39.6	22.9	59.1	32.5	17.5	52.2	13.1	4.4	33.3	37.0	20.4	57.3	44.2	26.1	63.9	33.8	17.8	54.6	37.0	20.4	57.3	41.1	23.6	61.1	27.0	12.8	48.1	40.1	23.0	60.0	34.5	21.5	47.6	29
**Locality**																																		
Urban	67.1	55.5	76.9	58.5	46.5	69.6	20.1	12.5	30.9	41.2	30.3	53.0	42.0	30.9	53.9	35.4	24.8	47.6	40.0	29.0	52.0	41.4	30.3	53.4	37.5	26.9	49.5	46.3	34.9	58.0	42.9	35.5	50.3	72
Rural	64.8	57.4	71.6	56.1	48.8	63.2	8.7	5.3	13.9	29.4	23.2	36.5	33.2	26.6	40.5	24.8	19.0	31.6	32.1	25.6	39.4	33.8	27.3	40.9	27.8	21.9	34.5	49.7	42.5	56.8	36.0	32.0	40.1	207
**Zone**																																		
North	66.3	52.0	78.1	72.2	58.3	82.9	21.0	11.9	34.4	38.0	25.7	52.0	40.1	27.6	54.0	35.0	23.1	49.1	37.9	25.8	51.8	37.9	25.8	51.8	35.8	23.9	49.7	59.9	45.6	72.7	44.4	36.1	52.7	63
Dakar	63.4	44.9	78.6	51.7	32.9	70.1	19.9	8.7	39.3	34.8	19.2	54.5	43.1	25.8	62.2	35.1	19.0	55.3	38.4	21.7	58.3	38.4	21.7	58.3	38.2	21.6	58.2	36.7	20.2	57.0	40.0	28.0	52.0	28
Thies	44.7	27.4	63.4	37.0	21.2	56.2	14.0	5.2	32.7	36.2	20.4	55.7	40.0	23.4	59.3	31.2	16.5	50.9	32.3	17.5	51.9	31.2	16.5	50.9	31.8	17.2	51.2	59.2	40.8	75.4	35.8	23.0	48.5	35
Central	66.6	55.0	76.4	48.6	37.3	60.1	7.5	3.5	15.4	42.2	31.3	54.0	42.8	31.9	54.4	34.1	24.4	45.4	39.6	29.0	51.4	46.5	35.9	57.4	35.3	26.8	44.9	43.7	32.4	55.8	40.7	34.7	46.7	79
East	70.2	50.2	84.6	64.7	44.6	80.7	0.0			15.2	6.0	33.6	17.4	7.3	36.2	13.9	5.6	30.2	29.9	15.9	49.1	25.7	12.6	45.4	25.3	11.7	46.4	30.7	18.7	46.2	29.3	20.2	38.4	29
South	76.5	60.7	87.3	62.7	46.9	76.2	6.2	2.0	17.7	14.5	8.0	24.9	19.6	10.6	33.4	7.1	2.1	21.4	20.9	11.8	34.4	22.4	13.0	36.0	11.3	4.9	23.8	52.7	39.3	65.7	29.4	23.4	35.4	46
**Total**	**65.4**	**59.3**	**71.1**	**56.7**	**50.6**	**62.7**	**11.6**	**8.2**	**16.2**	**32.5**	**27.1**	**38.4**	**35.5**	**29.8**	**41.5**	**27.5**	**22.4**	**33.3**	**34.1**	**28.5**	**40.2**	**35.7**	**30.2**	**41.7**	**30.3**	**25.2**	**36.0**	**48.8**	**42.8**	**54.8**	**37.8**	**34.3**	**41.3**	**279**

Note: LB and UB refer to the lower and upper bounds of the 95% confidence interval.
